# Remifentanil does not affect human microglial immune activation in response to pro-inflammatory cytokines

**DOI:** 10.17179/excli2022-5667

**Published:** 2023-02-24

**Authors:** Cinzia Dello Russo, Natalia Cappoli, Elisabetta Tabolacci, Liliana Sollazzi, Pierluigi Navarra, Paola Aceto

**Affiliations:** 1 Dipartimento di Sicurezza e Bioetica, Sezione di Farmacologia, Università Cattolica Del Sacro Cuore, Fondazione Policlinico Universitario A. Gemelli IRCCS, Rome, Italy; 2 Department of Pharmacology & Therapeutics, Institute of Systems Molecular and Integrative Biology (ISMIB), University of Liverpool, Liverpool, United Kingdom; 3 Dipartimento di Scienze Della Vita e Sanità Pubblica, Sezione di Medicina Genomica, Università Cattolica del Sacro Cuore, Fondazione Policlinico Universitario A. Gemelli IRCCS, Rome, Italy; 4 Dipartimento di Scienze dell'Emergenza, Anestesiologiche e della Rianimazione, Fondazione Policlinico Universitario A. Gemelli IRCCS, Rome, Italy; 5 Dipartimento di Scienze Biotecnologiche di Base, Cliniche Intensivologiche e Perioperatorie, Università Cattolica del Sacro Cuore, Rome, Italy

**Keywords:** human microglia, remifentanil, hyperalgesia, interleukin-6, interleukin-8, monocyte chemotactic protein 1

## Abstract

Remifentanil is a potent ultra-short acting μ-opioid analgesic drug, frequently used in anaesthesia due to its favorable pharmacodynamic and pharmacokinetic profile. It may be associated with the occurrence of hyperalgesia. Preclinical studies suggest a potential role of microglia, although the molecular mechanisms have not been fully elucidated. Considering the role of microglia in brain inflammation and the relevant differences among species, the effects of remifentanil were studied on the human microglial C20 cells. The drug was tested at clinically relevant concentrations under basal and inflammatory conditions*.* In the C20 cells, the expression and secretion of interleukin 6, interleukin 8 and the monocyte chemotactic protein 1 were rapidly induced by a mixture of pro-inflammatory cytokines. This stimulatory effect was sustained up to 24 h. Remifentanil did not exert any toxic effect nor modify the production of these inflammatory mediators, thus suggesting the lack of direct immune modulatory actions on human microglia.

## Abbreviations

BDNF brain-derived neurotrophic factor

CM complete medium 

CNS central nervous system

DH dorsal horn 

DRG dorsal root ganglia 

IL interleukin

IFNγ interferon-γ

LTP long-term potentiation

MCP-1 monocyte chemotactic protein 1

NMDA N-methyl-D-aspartate

OIH Opioid-induced hyperalgesia 

RF Remifentanil

RIH remifentanil induced hyperalgesia 

TII mixture of TNFα, IL-1β and IFNγ

TNFα tumor necrosis factor 

## Introduction

Remifentanil (RF) is a highly potent ultra-short acting μ-opioid analgesic drug (Yu et al., 2016[44]). It is frequently used in surgical anesthesia due to its favorable pharmacodynamic and pharmacokinetic profile. The drug is highly lipid soluble thus allowing for the fast onset of the pharmacological effects. It is rapidly metabolized by plasma and tissue non-specific esterases. Therefore, it is characterized by short half-life (3-5 min). The rapid elimination explains the fast recovery of the clinical effects observed at the end of RF infusion, and the lack of cumulative effects (Aceto et al., 2019[3]). However, the clinical benefits of RF may be hampered by development of acute opioid tolerance and/or RF-induced hyperalgesia (RIH). These conditions are associated with increased post-operative pain perception, thus augmented opioid consumption, patients' discomfort and prolonged hospital stay (Santonocito et al., 2018[35]). Acute opioid tolerance occurs few hours after the initiation of RF infusion. It can be managed by increasing RF dose during surgery and by administration of long-acting opioid bridging therapy at the end of the surgical procedures, to limit the extent of post-surgical pain (Niedermayer et al., 2020[29]). On the other hand, RIH is characterized by a paradoxical increase in pain intensity at the site of surgical wound (primary hyperalgesia), often associated with secondary hyperalgesia and allodynia (Yu et al., 2016[44]). RIH seems to occur predominantly after the administration of high intraoperative doses (Fletcher and Martinez, 2014[16]) and after prolonged infusions of RF, often required during extensive and/or long surgical procedures (Aceto et al., 2019[3]). Once established, this condition is difficult to treat. It is worsened by increasing opioid dose administration and may contribute to the development of chronic pain (Niedermayer et al., 2020[29]). 

The pathogenesis of RIH is not completely elucidated. It appears to be complex involving both peripheral and central mechanisms of neuronal sensitization (Santonocito et al., 2018[35]). Preclinical data show that RF can directly modulate the excitatory neuronal transmission, promoting the activation of the NMDA (N-methyl-D-aspartate) receptors, with subsequent increase in intracellular Ca^++^ influx, neuronal depolarization, and propagation of pain signals (Zhao and Joo, 2008[47]; Zheng et al., 2012[48]; Zhang et al., 2015[46]). In addition, the pro-nociceptive actions of RF seem to be mediated by the activation of other receptors and downstream signaling pathways, including the delta opioid receptors (Wang et al., 2015[40]) and the purinergic P2Y1 receptors (Su et al., 2021[39]). In different animal models, increased expression, and activity of the glutamate AMPA (α-amino-3-hydroxy-5-methyl-4-isoxazole propionic acid) receptors was found in association with RIH, both in the dorsal root ganglia (DRG) and in the dorsal horn (DH) of the spinal cord (Wang et al., 2020[41]; Li et al., 2021[26]). The activation of the AMPA receptors appeared dependent on the purinergic P2X4 receptor activation via the brain-derived neurotrophic factor (BDNF)/ TrkB signaling pathway (Fu et al., 2021[17]). Notably, the activation of both NMDA and AMPA receptors can increase the transmission of nociceptive signals. Moreover, RF may also affect the activity of the GABAergic system by different mechanisms, including reduction of GABA synthesis, downregulation of GABA receptors and the K^+^/Cl^-^-cotransporter 2 (KCC2), normally involved in the regulation of intracellular Cl^-^ concentrations (Gao et al., 2022[19]). Additional mechanisms that favor the development of RIH include the production of spinal dynorphins, the activation of descending facilitating fibres, and genetic factors (Yu et al., 2016[44]).

Interestingly, the role of glial cell activation in promoting peripheral and central sensitization was also hypothesized as a pathogenic mechanism underlying RIH. Preclinical studies showed the activation of satellite glial cells in the DRG and glial cells in the DH of the spinal cord during RF infusion (Romero et al., 2013[34]; Horii et al., 2020[23]). A recent study suggests a complex interplay between glial cells and neurons in the spinal cord in response to RF. In particular, RF promoted the activation of the NLRP3 inflammasome, with increased production of interleukin (IL)-1β, increased phosphorylation of the NR1 subunit of the neuronal NMDA receptors, and downregulation of the glutamate transporter GLT1 in astrocytes (Yuan et al., 2022[45]). Increased activation of microglial cells together with augmented release of tumor necrosis factor α (TNFα) was also detected in response to RF withdrawal, leading to long-term potentiation (LTP) at the C-fibre synapses. The latter is a well-characterized mechanism underlying synaptic plasticity and development of hyperalgesia in rats. In this setting, protective actions of minocycline, a known microglial inhibitor drug, were observed (Yang et al., 2018[42]). However, a previous study failed to demonstrate the beneficial effects of minocycline on RIH in rats (Aguado et al., 2015[4]). It is possible that the 'opioid-withdrawal LTP' at the C-fibre synapses also requires the activation of astrocytes in the spinal cord (Drdla-Schutting et al., 2019[15]). On the other hand, recent *in vitro *data suggest potential anti-inflammatory actions of RF on microglial activation. The drug was indeed able to reduce the production of IL-6, TNFα, the expression and activity of the inducible nitric oxide synthase and cyclooxygenase 2 in the murine microglial BV2 cell line activated with the bacterial endotoxin, lipopolysaccharide (Huang et al., 2022[24]). In line with these data, RF reduced the release of pro-inflammatory cytokines, such as IL-8, IL-6 and TNFα, in combined anesthesia in humans (Ding et al, 2019[14]). In this scenario, it is likely that microglial cells may play a role in RIH, but the issue needs further investigation. Moreover, considering the important differences in the immune response among different species (Smith and Dragunow, 2014[38]), it is crucial to characterize the effects of RF in experimental models of human microglia.

In a previous study (Cappoli et al., 2021[7]), we used the human microglial C20 cell line (Garcia-Mesa et al., 2017[20]) to test the hypothesis that RF could modulate microglial activation, thus contributing to development of inflammation in the central nervous system (CNS) and hyperalgesia in humans. The C20 microglial cells express the μ-opioid receptor (Cappoli et al., 2021[7]), hence supporting the hypothesis that RF could exert direct modulatory actions on human microglia. However, when tested at 5 ng/ml in 16 hour-experiments, the drug did not modulate neither the secretion nor the expression of IL-6 in the C20 cells. The lack of RF modulatory effects was observed both in basal and inflammatory conditions (Cappoli et al., 2021[7]). Considering the potential involvement of the BDNF pathway in pain transmission and chronic pain syndromes (Cappoli et al., 2020[9]), we also evaluated the effect of RF on human microglial BDNF production. In this regard, mature BDNF was undetectable in the incubation medium under basal conditions, nor its secretion was significantly increased after inflammatory microglial activation. Notably, RF increased BDNF mRNA transcription and tended to augment the intracellular levels of BDNF, albeit no differences in the secretion were detected thus suggesting potential pro-nociceptive action of RF through this pathway (Cappoli et al., 2021[7]). 

In the present study, we further expanded these initial observations, focusing on other microglial inflammatory mediators with a prominent role in the initiation and maintenance of CNS inflammatory processes. We further characterized the response of the C20 cells to inflammatory cytokines, namely TNFα, IL-1β and IFNγ (TII). TII promotes a rapid increase in the secretion of different inflammatory markers, including IL-6, the monocyte chemotactic protein 1 (MCP-1) and IL-8. The aim of this study was to investigate if RF, at clinically relevant concentrations, could modulate the expression and secretion of these additional markers of microglial immune activation, thus leading to hyperalgesia. 

## Materials and Methods

### Materials

The human microglial C20 cell line was kindly provided by Dr Álvarez-Carbonell, (Case Western Reserve University, Cleveland, OH, USA) (Garcia-Mesa et al., 2017[20]). The BrainPhys culture medium (cat. n. 5791) and the N2 supplement (cat. n. 7152) were from StemCell Technologies (Vancouver, Canada). Fetal Bovine Serum (FBS, cat. n. S0115) was purchased by Biochrom AG (Berlin, Germany). Antibiotics, namely penicillin/streptomycin (cat. n. A2213), were from Biochrom (Berlin, Germany) and normocin (cat. n. ant-nr-1) was from Invivogen (San Diego, CA, USA). The Hanks Balanced Salt Solution (HBSS) was from Gibco (Thermo Fisher Scientific, Waltham, MA, USA). The recombinant pro-inflammatory cytokines, namely the human TNFα (cat. n. RTNFAI) and the human IL-1β (cat. n. RIL1BI) were from Invitrogen (Thermo Fisher Scientific). The human interferon-γ (IFNγ, cat. n. SRP3058) was purchased by Sigma-Aldrich (Saint Louis, MO, USA). Cytokines were reconstituted in certified endotoxin-free water (G-Biosciences, St. Louis, MO, USA) and further diluted as previously described (Cappoli et al., 2019[8]). A mixture of TNFα, IL-1β and IFNγ (TII) was used as pro-inflammatory stimulus. Each cytokine was diluted at the same concentration, *i.e.*, 50 pg/ml, in the cocktail. The latter is the optimal concentration to increase the release of IL-6 in this experimental model (Cappoli et al., 2021[7]). 

### Cell cultures and RF treatment

The C20 cell line was developed through SV40/hTERT immortalization of primary cultures of human microglia derived from the cortical tissue of adult subjects (Garcia-Mesa et al., 2017[20]). RNA-seq analysis showed that the C20 clone retains the genetic signature of primary human microglia. Cells were maintained according to the protocol provided by Dr Álvarez-Carbonell, optimized to preserve the specific microglial phenotype *in vitro*. Briefly, cells were cultured in complete medium (CM), consisting of BrainPhys medium supplemented with 1 % N2, 1 % FBS, and antibiotics (100 IU/mL of penicillin, 100 μg/mL of streptomycin and 100 μg/mL of normocin). Cells were plated at the density of 20,000 cells/cm^2^ in 75 cm^2^ flasks and incubated at 37 °C in a humidified atmosphere containing 5 % CO_2_. The incubation medium was replaced the day after plating and every three or four days. Cells were passaged once or twice a week, when they reached 90-95 % confluency. The identity of the C20 cells was verified by confirming the expression of specific microglial lineage markers, namely the ionized calcium-binding adaptor protein-1, the C-X3-C Motif Chemokine Receptor 1, the purinergic receptor P2RY12; the transmembrane protein 119 and the colony stimulating factor 1 receptor (Cappoli et al., 2021[7]). All these evaluations were performed using specific primers, which can detect only human amplicons of these transcripts. In addition, taking the expression and secretion of IL-6 as a readout of microglial inflammatory activation, we first confirmed that the C20 cells could be activated in response to 10 pg/ml TNFα in line with the original characterization (Garcia-Mesa et al., 2017[20]). For functional experiments, cells were plated at 60,000 cells/cm^2^, let to recover overnight and stimulated the day after in CM. RF (Ultiva, Aspen Pharma Trading Ltd, Dublin, Ireland) was dissolved in saline buffer at 50 µg/ml, further diluted in CM and tested alone or in combination with TII, at different time points.

### IL-6, IL-8 and MCP-1 measurements

The levels of IL-6, IL-8 and MCP-1 in the incubation medium were determined by specific DuoSet ELISA development system assays (R&D Systems, Minneapolis, MN, USA), according to the manufacturer's instructions. Briefly, aliquots of 90 μl of incubation medium were assayed for the Control groups, *i.e.*, microglial cells incubated in CM for different times. On the other hand, we used aliquots of different volumes for the TII-treated samples in order to measure the optical density in the linear range of the standard curve. The selected volumes were 25 μl for IL-6, 4 or 20 μl for IL-8, and 4, 10, or 20 μl for MCP-1 depending on the incubation time point measured. A standard curve was generated within the concentration ranges of 9.38 - 600 pg/ml for IL-6; 7.8 - 2000 pg/ml for IL-8 and 15.6 - 1000 pg/ml for MCP-1. The optical density of each well was quantified using a microplate reader (Victor-X^TM^4, PerkinElmer Inc) set to 450 nm with wavelength correction to 570 nm. A net optical density value was calculated for each standard and sample, by subtracting the absorbance reading values of the zero standards or blanks (plain medium not exposed to the cells), respectively. The standard curve for each cytokine was generated through a four-parameter logistic curve-fit.

### Cell viability and cytotoxicity

Microglial viability was measured by the CellTiter AQueous One Solution Reagent (cat. n. G3582) (Promega, Madison, WI, USA), as previously described (Cappoli et al., 2019[8]). The toxicity induced by RF alone or in combination with TII was assessed by measuring the lactate dehydrogenase (LDH) activity in the medium through the CytoTox 96 kit (cat. N. G1780, Promega). Briefly, 50 μL of cell culture medium were incubated with 50 μL of CytoTox 96 Reagent, at room temperature for 30 min. Samples were protected from the light. The reaction was stopped by the addition of 50 μl of Stop Solution. Blanks consisted in 50 μL of culture medium not exposed to the cells. The absorbance at 490 nm was recorded by using a spectrophotometer plate reader (iMark, Biorad, Hercules, CA, USA).

### mRNA analysis in real time PCR

Expression of inflammatory genes was quantified through real-time qPCR, as previously described (Dello Russo et al., 2009[13]). Briefly, total RNA was extracted from cells using TRIZOL reagent (Invitrogen) and treated with the DNAse-free^TM^ Kit (Ambion, Life Technologies, cat. n. AM1906), according to the manufacturer's instructions. Shortly, total RNA was incubated with DNAse for 30 min, then resuspended in DNAse inactivation reagent. The RNA concentration was quantified, and aliquots (1 µg) were converted to cDNA using random hexamer primers and the ImProm-II Reverse Transcriptase kit (Promega). The real-time qPCRs were performed using the Brilliant SYBR Green QPCR Master Mix 2X (Agilent) and the following cycling conditions: 35 cycles of denaturation at 95 °C for 20 s; annealing at 60 °C for 30 s; extension at 72 °C for 30 s. The primers used for the detection of gene expression were as follows: for *IL-6* (NM_000600.4), F394 (CCTTCCAAAGATGGCTGAAA) and R543 (TGGCTTGTTCCTCACTACT), which yield a 150 bp amplicon (Cappoli et al., 2019[8]); for *MCP-1* (NM_002982.3), F199 (GATCTCAGTGCAGAGGCTCG) and R351 (TGCTTGTCCAGGTGGTCCAT), which yield a 153 bp amplicon; for *IL-8* (NM_000584.3), F110 (CCAGGAAGAAACCACCGGA) and R220 (GAAATCAGGAAGGCTGCCAAG), which yield a 111 bp amplification product; for *ACTB* (β-actin) (NM_001101.4), F427(TGGGACGACATGGAGAAA) and R573 (GAAGGTCTCAAACATGATCTGG), which yield a 147 bp amplicon (Cappoli et al., 2019[8]). The real-time qPCR reactions were carried out in 20 μl reaction volume in a MX3000P real-time PCR machine (Stratagene). Relative mRNA concentrations were calculated from the take-off point of reactions (threshold cycle, Ct) using the comparative quantitation method (Dello Russo et al., 2009[13]). Ct values for the *ACTB* gene expression were used as a normalizing signal. *ACTB* was selected considering that it is constitutively expressed in human microglia at high level (Gosselin et al., 2017[21]). The efficiency of the real-time qPCRs ranged between 96 and 107.9 %. At the end of amplification, the products were separated by electrophoresis through 2 % agarose gels containing 0.1 μg/ml ethidium bromide to ensure the product was correct in size.

### Data analysis

Experiments for the evaluation of cytokines release and treatment toxicity were carried out using 4-5 biological replicates per each experimental group and repeated at least 3 times. For the mRNA analysis, we used one biological replicate per each treatment and the experiments were repeated at least 3 times. Each sample was assayed in triplicates (technical replicates). All data were analyzed by one- or two-way ANOVA followed by Bonferroni's post hoc test. The level of significance was set at p ≤ 0.05. Data analysis was performed using GraphPad Prism 5 software (San Diego, CA, USA).

## Results

### Effects of pro-inflammatory stimulation on cytokines and chemokines expression and release 

**Figure 1:**
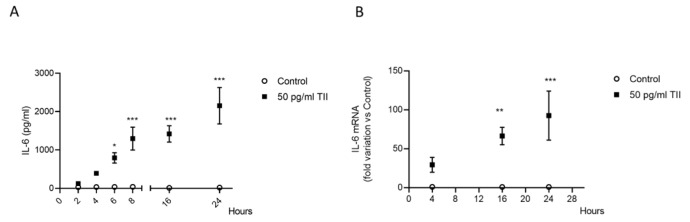
TII stimulatory effects on IL-6 expression and secretion. (A) IL-6 release was measured by a specific ELISA. Cells were treated with TII for different time points and the incubation medium was harvested and analyzed. (B) C20 cells were plated and treated with TII for different times. mRNA levels were evaluated taking the Control as calibrator. The experiment was repeated at least three times with similar results. Data are shown as pg/ml, mean ± SEM (n=11-12) or expressed as fold variation vs. Control, means ± SEM (n=11-12). Data were analyzed by two-way ANOVA followed by the Bonferroni's *post hoc *test. *, p<0.05; **, p<0.01; and ***, P<0.001, versus Control

In our previous study, we have shown that 50 pg/ml TII significantly increases the expression and secretion of IL-6 by the human C20 microglial cells after 16 h (Cappoli et al., 2021[7]). To further characterize the kinetics of IL-6 production in this experimental model, the C20 cells were treated with 50 pg/ml TII for different times, from 2 to 24 h. The levels of IL-6 released in the incubation media were assessed. The C20 cells produced sizable amounts of IL-6 under basal conditions, *i.e.*, 29.19 pg/ml ± 2.71 (mean ± SEM, n=12) after 2 h of incubation in CM (Table 1(Tab. 1)). The secretion of IL-6 tended to be stable over time, with a mean production of 27.68 ± 1.86 pg/ml (mean_2h-24h_ ± SEM, n=6, Table 1(Tab. 1)). As shown in Figure 1A(Fig. 1), TII augmented the release of IL-6 in the incubation media, with a significant effect observed after 6 h of incubation. The secretion of IL-6 in the incubation medium was sustained over time, with maximal stimulatory effects observed at 24 h, the later time point studied (Figure 1A(Fig. 1)). Data are summarized in Table 1(Tab. 1). The release of IL-6 was paralleled by the upregulation of the IL-6 mRNA, which was significant after 4 h of incubation with TII and continued to increase up to 24 h (Figure 1B(Fig. 1)).

**Figure 2:**
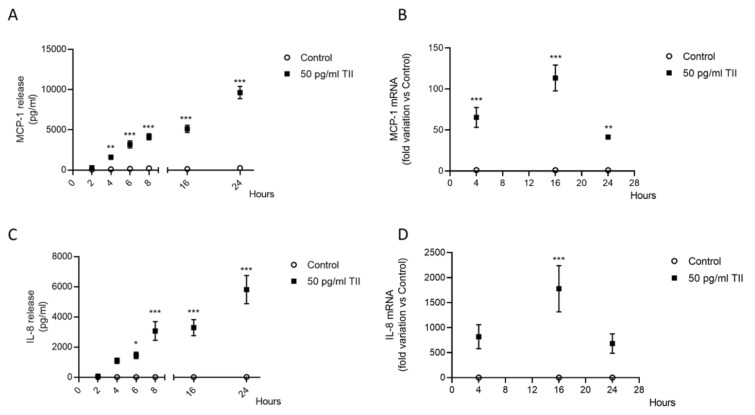
TII stimulatory effects on MCP-1 and IL-8 expression and secretion. (A) MCP-1 release was measured by a specific ELISA. Cells were treated for different time points and the incubation medium was harvested and analyzed. (B) C20 cells were plated and treated with TII for different times. mRNA was evaluated taking the Control as calibrator. The experiments were repeated at least three times with similar results. MCP-1 data are shown as pg/ml, mean ± SEM (n=11-12) or expressed as fold variation vs. Control, means ± SEM (n=10-12). Data were analyzed by two-way ANOVA followed by the Bonferroni's *post hoc *test. **, p<0.01; and ***, P<0.001, versus Control. (C) IL-8 release in the incubation medium was measured by a specific ELISA, after different time points. (D) IL-8 mRNA was evaluated taking the Control as calibrator after C20 cell treatment with TII for different times. The experiments were repeated at least three times with similar results. Data are shown as pg/ml, mean ± SEM (n=15-16) or expressed as fold variation vs. Control, means ± SEM (n=7-12). Data were analyzed by two-way ANOVA followed by the Bonferroni's *post hoc *test. *, p<0.05; and ***, p<0.001, versus Control

A preliminary proteomic analysis showed that TII significantly increased the release of other inflammatory factors in long-term incubation experiments, including MCP-1 and IL-8 (Cappoli et al., 2021[7]). In line with these initial observations, we found that TII significantly enhanced the release on MCP-1 in the incubation medium, even after shorter incubation times (4 h) in comparison to IL-6 (Figure 2A(Fig. 2)). As shown in Table 1(Tab. 1), MCP-1 was produced at a higher level than IL-6 both under basal conditions and in response to TII, at the different time-points analyzed. The release of MCP-1 tended to increase over time both under basal conditions and in presence of TII, with maximal levels measured at 24 h (Table 1(Tab. 1), Figure 2A(Fig. 2)). Likewise, the release of MCP-1 was paralleled by the upregulation of the mRNA levels, which was significant after 4 h of incubation and reached maximal levels after 16 h (Figure 2B(Fig. 2)). In addition, the C20 cells produced sizable amounts of IL-8 under basal conditions, *i.e.*, 11.38 pg/ml ± 0.95 (mean ± SEM, n=16) after 2 h of incubation in CM (Table 1(Tab. 1)). The secretion of IL-8 appeared stable over time, with a mean production of 17.09 ± 0.71 pg/ml (mean_2h-24h_ ± SEM, n=6, Table 1(Tab. 1)). We observed similar kinetics between IL-8 and MCP-1 in response to TII, with respect to both secretion (Figure 2C(Fig. 2)) and mRNA expression (Figure 2D(Fig. 2)). However, the release of IL-8 resulted significantly incremented after 6 h of incubation in response to TII, similarly to IL-6 (Figure 2C(Fig. 2)). Notably, IL-8 was produced at higher levels than IL-6 after stimulation with TII (Table 1(Tab. 1)). Taken together these data suggest that the human C20 microglial cells respond to TII with increased release of several inflammatory markers, including MCP-1, the factor most abundantly produced and rapidly upregulated.

### Effects of RF on human microglial cell viability and immune activation

**Figure 3:**
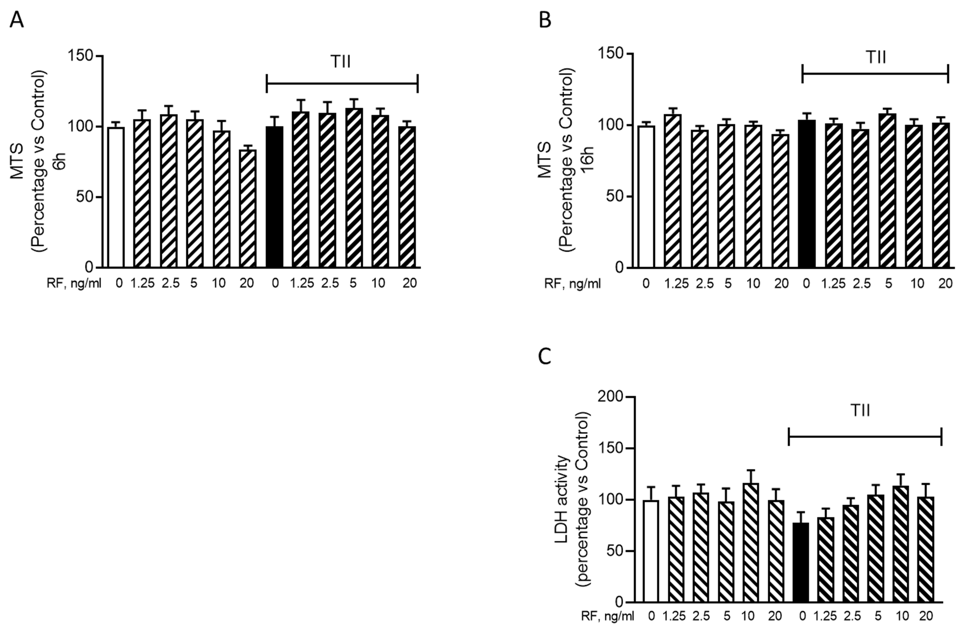
Effects of remifentanil on cell viability and cytotoxicity. (A-B) Cell viability was detected by MTS reduction assay at two different time points, 6 h (A) and 16 h (B). C20 cells were treated with remifentanil (RF) alone at different concentrations and in combination with 50 pg/ml TII. Results are shown as fold changes versus Control, set as 100 %. Results are pooled analysis of three independent experiments. Data are means ± SEM (n=14) and were analyzed by one-way ANOVA followed by the Bonferroni's *post hoc *test. (C) The cytotoxic effects of the treatments were assessed by measuring the release of LDH in the incubation medium after 16 h incubation, taken as an index of cell toxicity. None of the treatments was toxic for C20 microglial cells. Results are shown as fold changes versus Control, whose optical density was set as 100 %. Results are pooled analysis of three independent experiments. Data are means ± SEM (n=12-14) and were analyzed by one-way ANOVA followed by the Bonferroni's *post hoc *test.

When tested in the range of clinically relevant concentrations (1.25-20 ng/ml) (Aceto et al, 2017[2]), RF did not exert any toxic effect on C20 cells. Cytotoxic effects were excluded by assessing cell viability, via reduction of the MTS tetrazolium compound after short-term (6 h) and long-term (16 h) incubations both under basal and inflammatory conditions (Figure 3A-B(Fig. 3)). In addition, measurements of LDH activity in the incubation media at the end of 16 h treatments confirmed the lack of cytotoxic effects of RF in this experimental model (Figure 3C(Fig. 3)).

**Figure 4:**
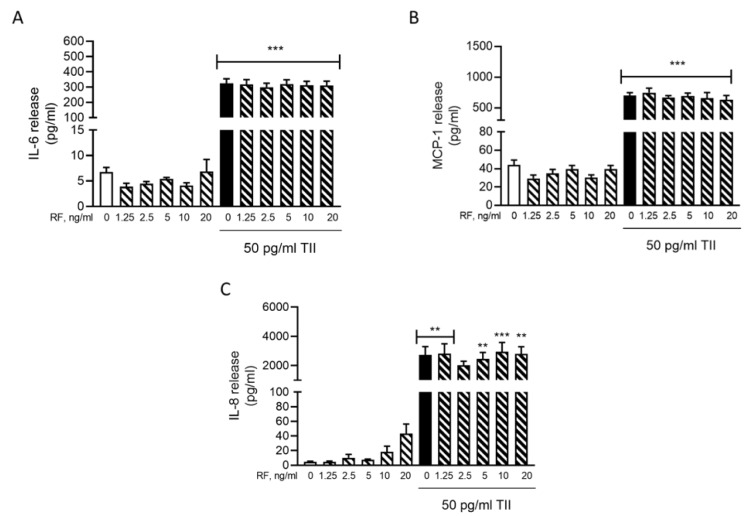
Effects of remifentanil on IL-6, MCP-1 and IL-8 release. C20 cells were treated with remifentanil (RF) at different concentrations alone or in combination with 50 pg/ml TII for 6 h. (A) The incubation medium was used to evaluate the release of IL-6 with a specific ELISA. Results are pooled analysis of three independent experiments. Data are means ± SEM (n=7-10) and were analyzed by one-way ANOVA followed by the Bonferroni's post hoc test. ***, P<0.001, versus Control. (B) The incubation medium was used to evaluate the release of MCP-1 with a specific ELISA. Results are pooled analysis of three independent experiments. Data are means ± SEM (n=11-12) were analyzed by one-way ANOVA followed by the Bonferroni's post hoc test. ***, P<0.001, versus Control. (C) The incubation medium was used to evaluate the release of IL-8 with a specific ELISA. Results are pooled analysis of three independent experiments. Data are means ± SEM (n=8-16) and were analyzed by one-way ANOVA followed by the Bonferroni's post hoc test. **, p<0.01; ***, p<0.001, versus Control

**Figure 5:**
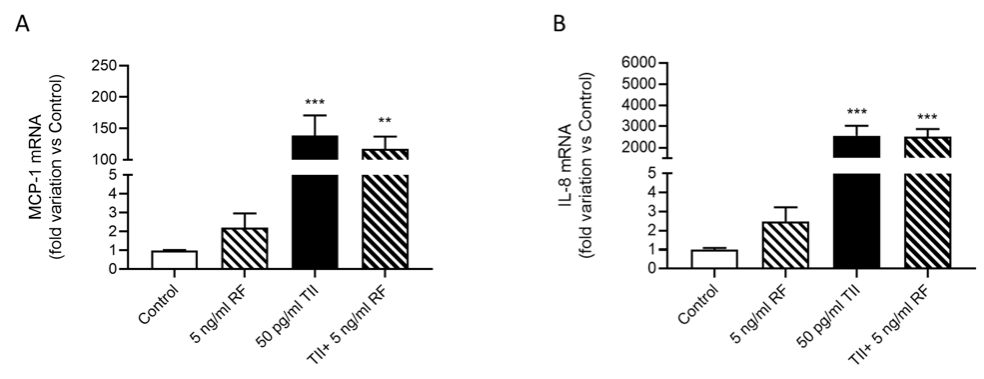
Effects of remifentanil (RF) on IL-8 and MCP-1 expression. C20 cells were treated with 5 ng/ml RF alone or in combination with 50 pg/ml TII for 16 h. (A) MCP-1 and (B) IL-8 mRNA levels were assessed by real-time qPCR. Results are pooled analysis of three independent experiments. Data are expressed as fold variation vs Control, means ± SEM (n=7-9) and were analyzed by one-way ANOVA followed by the Bonferroni's post hoc test. **, p<0.01; ***, p<0.001, versus Control

To determine the effects of RF on microglial inflammatory activation, we evaluated the release of IL-6 (Figure 4A(Fig. 4)), MCP-1 (Figure 4B(Fig. 4)) and IL-8 (Figure 4C(Fig. 4)) in the C20 cells treated with RF alone or in combination with 50 pg/ml TII, for 4 h or 6 h. The time point for these evaluations was selected based on the results of the time-course experiments (Figure 1A(Fig. 1) for IL-6, Figure 2A(Fig. 2) for MCP-1 and Figure 2C(Fig. 2) for IL-8). We selected the shortest time at which a significant stimulatory effect of TII on these inflammatory mediators was observed. In the concentration range between 1.25-20 ng/ml, RF did not modify the release of these inflammatory markers neither under basal conditions nor in cells activated with TII (Figure 4A-C(Fig. 4)). However, a relatively high number of undetectable (ND) readings was observed in some experiments carried out to assess the release of IL-6 (Supplementary Table 10) and IL-8 (Supplementary Table 12). These results may reflect a different degree of basal activation of the C20 cells, in line with the intrinsic variability of the cell system (Supplementary Tables 1, 3, 5). For this reason, the basal production of these cytokines may result below the detection limit of the assay in certain experiments. Notably, RF did not increase the basal release of both IL-6 and IL-8 in these experiments, further confirming the lack of pro-inflammatory actions in human microglia. On the other hand, it was unexpected to observe ND values for the measure of IL-6 in TII stimulated cells (Supplementary Table 10). Although this may represent a limit of our study, we need to consider that the average concentration of IL-6 measured in TII treated samples, excluding the ND values, was in line with all previous experiments (Supplementary Tables 1 and 10, experiment n. 1). When tested at 5 ng/ml in 16 h experiments, RF did not modulate neither the secretion nor the expression of IL-6 in C20 cells, in basal and inflammatory conditions (Cappoli et al., 2021[7]). The RF concentration (5 ng/ml) tested in these long-term experiments was selected according to the mean plasma concentration detected in patients undergoing surgical anesthesia (Aceto et al., 2017[2]). In line with the lack of modulation of IL-6 mRNA, 5 ng/ml RF did not modify the expression levels of both MCP-1 and IL-8, neither under basal nor under inflammatory conditions (Figure 5A-B(Fig. 5)).

## Discussion

In the present study, we expanded our initial observations on the effects of RF in the modulation of human microglial inflammatory activation (Cappoli et al., 2021[7]). We further characterized the response of the C20 cells to TII studying the effects of these cytokines on the production of IL-6 over time (2 h - 24 h). We have previously shown that IL-6 is readily upregulated in response to a pro-inflammatory stimulation in the human embryonic microglial HMC3 cells (Dello Russo et al., 2018[12]; Cappoli et al., 2019[8]), thus it can be considered a reliable inflammatory marker for human microglia. TII is able to significantly increase the release of IL-6 after 6 h of incubation, with further increments observed at the later time points up to 24 h. The secretion of IL-6 was sustained by the mRNA upregulation, with a significant increase observed after 4 h and levels that continue to increase up to 24 h. In line with our previous data, RF tested in a wide range of clinically relevant concentrations (Aceto et al., 2017[2]) was not able to modulate IL-6 release neither under basal conditions nor in combination with TII, in short-term experiments, *i.e.,* 6 h of incubation. 

Notably, a preliminary proteomic analysis revealed an increased release of other inflammatory factors, besides IL-6, in response to TII. These markers included MCP-1, IL-8 and the C-X-C Motif Chemokine Ligand (CXCL) 1 (CXCL1). Interestingly, the secretion of MCP-1 and IL-8 appeared to be higher than the amounts of IL-6 released in the incubation media (Cappoli et al. 2021[7]). These results are in line with the original characterization of the C20 cells. MCP-1 was produced at a higher level compared to IL-6 and IL-8 under basal conditions (Garcia-Mesa et al., 2017[20]). In addition, the cells responded to TNF-α (10 pg/mL, for 16 h) by increased secretion of IL-8 at a high level and production of MCP-1 and IL-6 at medium level (fold variation *versus* control) (Garcia-Mesa et al., 2017[20]). Moreover, it has been shown that the C20 cells respond to 20 ng/ml IL-1β with increased production of IL-6 and chemokines, including CCL2/MCP-1 and CXCL10/IP-10 (Davis et al., 2018[11]; Pozzo et al., 2019[30]). In addition, IL-1β at 20 ng/ml alone or at 100 ng/ml in combination with 50 ng/ml IFNγ was able to increase the expression level of IL-8 after 24 h treatment (Pozzo et al., 2019[30]). Interestingly, the proteome profiler assay used for our initial experiments with the C20 cells is a membrane-based antibody array. This assay allows for the simultaneous evaluation of the relative quantity of selected human cytokines and chemokines. We could rapidly evaluate the release of 36 pro- and anti-inflammatory molecules in control conditions and after a challenge with TII alone or in combination with 5 ng/ml RF for 20 h. However, this evaluation was performed in a single sample per treatment. The results obtained with this approach should therefore be considered as a guide for subsequent investigations. In fact, we were able to confirm the stimulatory effects of TII on both MCP-1 and IL-8 over an extended time course, from 2 h up to 24 h, with specific ELISA assays. These data further suggest that the human microglial response to the inflammatory stimulation with TII is more complex than the simple release of IL-6 (Cappoli et al, 2021[7]). In our experimental conditions, we observed a more marked production of chemokines, like MCP-1 and IL-8, when compared to the release of pro-inflammatory IL-6 in response to TII. This observation may suggest that, when exposed to inflammatory cytokines, microglial cells release relevant factors for the recruitment of peripheral immune cells and the maintenance of inflammatory processes in the central nervous system (CNS). Notably, MCP-1 and IL-8 are important chemokines for the recruitment of peripheral immune cells within inflamed tissues (Semple et al., 2010[36]; Revathikumar et al., 2016[31]). Indeed, IL-8 is known to induce neutrophil transmigration both *in vivo* and *in vitro *(Semple et al., 2010[36]). On the other hand, MCP-1 was the first chemokine to be characterized for the ability to activate and attract mononuclear cells, such as monocytes, macrophages and microglia, to inflamed sites (Yoshimura et al. 1989[43]; Rollins, 1996[33]). Similarly, the overexpression of MCP-1 in the CNS of transgenic mice promoted the accumulation of macrophages in the brain, further supporting the chemotactic functions of this chemokine (Fuentes et al., 1995[18]; Gunn et al., 1997[22]). Interestingly, MCP-1 can alter nociception; indeed, the administration of this chemokine can induce mechanical allodynia and neuropathic pain in CCR2-deficient mice (Abbadie et al., 2003[1]; Mélik-Parsadaniantz and Rostène, 2008[28]). 

On the other hand, the preliminary proteomic analysis revealed that RF at 5 ng/ml enhanced the secretion of MCP-1, IL-8 and CXCL1 in response to TII whereas completely reduced the amount of CXCL12 (Cappoli et al., 2021[7]). However, when tested over an extended range of concentrations (1.25 and 20 ng/ml) in short-term experiments, RF did not exert any relevant modulatory action on the production of both MCP-1 and IL-8. In addition, RF did not modulate the expression of MCP-1 and IL-8 after 16 h incubations. These data further suggest the lack of direct effects of RF in the modulation of microglial immune activation. Similarly, the proteomic approach showed a modest stimulatory effect of RF on IL-6, although the result was not confirmed by the specific ELISA (Cappoli et al., 2021[7]). Notably, RF did not exert any toxic effects on microglial cells. Taken together these data suggest that RF does not have any significant and direct pro-inflammatory effect on human microglial cells. This observation is particularly relevant considering that the release of pro-inflammatory cytokines, including IL-6, by peripheral immune cells was reported as a pathological mechanism underlying the development of RIH and other OIH (opioid-induced hyperalgesia) syndromes (Angst and Clark, 2006[5]; Comelon et al., 2016[10]; Sklika et al., 2016[37]; Aceto et al., 2019[3]). 

A pathogenic role of microglia in the development of opioids tolerance and OIH has been described using different preclinical experimental models (Roeckel et al., 2016[32]; Leduc-Pessah et al., 2017[25]). In this regard, the inhibition of microglial activation by various means seemed to be consistently associated with the reduction of hyperalgesia induced by morphine (Roeckel et al., 2016[32]). However, a recent paper demonstrated that the genetic ablation of microglial cells does not affect the occurrence of hyperalgesia associated with sustained morphine administration in mice (Liu et al., 2022[27]). Therefore, it is possible that different molecular as well as cellular mechanisms are activated in response to different opioids, doses or schedules of treatment, during the development of hyperalgesia. 

Interestingly, it has been recently shown that the withdrawal of RF after a short (60 min) infusion period, at a high dose (30 μg/kg in bolus followed by 450 μg/kg/h) in rats induced LTP at the c-fibre synapses in the spinal cord (Drdla-Schutting et al., 2019[15]). This effect was reduced by the direct application in the spinal cord of the non-selective glial toxin, fluoroacetate, and the microglial inhibitor, minocycline. However, the potential pro-nociceptive effects of microglial cells in this experimental model were not mediated by the release of known inflammatory mediators such as IL-1β and TNFα but required the production of D-serine at spinal level. The latter is a neuroactive amino acid with a pivotal role in NMDA receptor activation. In addition to their immune functions, microglial cells exert an important role in other cerebral processes, including the regulation of synaptic architecture and neurogenesis (Augusto-Oliveira et al., 2022[6]), which may be relevant to neuronal plasticity and sensitization. Therefore, it is possible that RF can modulate the production of neuroactive factors in human microglial cells, despite the lack of effects on microglial immune activation. Furthermore, it can be hypothesized that a potential increase in microglial activity during and/or after RF infusion in humans may be secondary to the neuronal activation along the pain transmission pathways and not directly related to the action of RF on these cells. 

In conclusion, RF showed neither toxic effects on microglial cells nor modulatory actions on the production of different inflammatory mediators. Taken together these data suggest that RF, at clinically relevant concentrations, is not able to directly modulate the immune activation of human microglia. Even if these results may be difficult to extrapolate to the clinical setting, this study could highlight the possibility that RIH could be mediated by alternate non-immune mediated central or peripheral mechanisms in humans. Our data do not support the development of therapeutic strategies aimed at modulating the immune responses of microglia in order to counteract RIH. 

## Declaration

### Conflict of interest

The authors declare that they have no conflict of interest.

### Acknowledgments

The authors thank Dr David Álvarez-Carbonell, Case Western Reserve University, Cleveland, OH, USA, for sharing the C20 cell line. The study was in part supported by UCSC Research Funds (D1-2017 to CDR, D1-2020 to LS, D1-2020 to ET) and by MIUR-PRIN Grant (201789LFKB) awarded to ET. 

## Supplementary Material

Supplementary data

## Figures and Tables

**Table 1:**
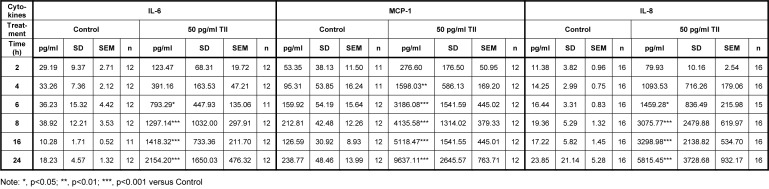
Release of cytokines by C20 cells stimulated with TII for different times (2 h-24 h)
