# Supplementary material for: Remifentanil does not affect human microglial immune activation in response to pro-inflammatory cytokines
Source: EXCLI J. 2023 Feb 24;22:295–309. doi: 10.17179/excli2022-5667 (PMC10201013; doi:10.17179/excli2022-5667)
Supplement: Supplementary data [file EXCLI-22-295-s-001.pdf]

EXCLI Journal 2023;22:295-309 – ISSN 1611-2156
Received: December 06, 2022, accepted: February 20, 2023, published: February 24, 2023

Supplementary data to:

Original article:

REMIFENTANIL DOES NOT AFFECT HUMAN MICROGLIAL IMMUNE ACTIVATION IN RESPONSE TO PRO-INFLAMMATORY CYTOKINES

Cinzia Dello Russo[1,2], Natalia Cappoli[1]*, Elisabetta Tabolacci[3], Liliana Sollazzi[4,5], Pierluigi Navarra[1], Paola Aceto[4,5]

[1] Dipartimento di Sicurezza e Bioetica, Sezione di Farmacologia, Università Cattolica Del Sacro Cuore, Fondazione Policlinico Universitario A. Gemelli IRCCS, Rome, Italy
[2] Department of Pharmacology & Therapeutics, Institute of Systems Molecular and Integrative Biology (ISMIB), University of Liverpool, Liverpool, United Kingdom
[3] Dipartimento di Scienze Della Vita e Sanità Pubblica, Sezione di Medicina Genomica, Università Cattolica del Sacro Cuore, Fondazione Policlinico Universitario A. Gemelli IRCCS, Rome, Italy
[4] Dipartimento di Scienze dell'Emergenza, Anestesiologiche e della Rianimazione, Fondazione Policlinico Universitario A. Gemelli IRCCS, Rome, Italy
[5] Dipartimento di Scienze Biotecnologiche di Base, Cliniche Intensivologiche e Perioperatorie, Università Cattolica del Sacro Cuore, Rome, Italy

* **Corresponding author:** Natalia Cappoli, PhD; Dipartimento di Sicurezza e Bioetica, Sezione di Farmacologia, Università Cattolica Del Sacro Cuore, L.go F Vito 1, 00168 Rome, Italy; E-mail: natalia.cappoli@unicatt.it

https://dx.doi.org/10.17179/excli2022-5667

This is an Open Access article distributed under the terms of the Creative Commons Attribution License (http://creativecommons.org/licenses/by/4.0/).

EXCLI Journal 2023;22:295-309 – ISSN 1611-2156
Received: December 06, 2022, accepted: February 20, 2023, published: February 24, 2023

Supplementary Table 1: Raw data concerning Figure 1A and Table 1. C20 microglial cells were stimulated with a mixture of proinflammatory cytokines, including TNFα, IL-1β and IFNγ (TII), each used at the same concentration of 50 pg/ml in the cocktail. Cells were treated for different times, from 2 h up to 24 h. After the incubation period, the concentration of interleukin 6 (IL-6, pg/ml) in the incubation medium was measured by a specific ELISA assay. The amounts of IL-6 measured in the incubation medium after different times of incubation are reported in this table. Data are shown as pg/ml and were obtained from 3 independent experiments, each including 4 biological replicates per treatment. ND = not detectable. The ND values were excluded from the analysis.

Treatments	Experiment 1				Experiment 2				Experiment 3			
	IL-6 concentration (pg/ml)				IL-6 Concentration (pg/ml)				IL-6 Concentration (pg/ml)			
**Control (2 h)**	22.16	26.85	22.23	18.35	17.67	19.03	33.70	33.86	42.05	32.94	37.45	44.02
**TII (2 h)**	51.46	79.32	64.09	36.15	68.17	62.44	161.75	183.05	166.49	188.25	218.53	201.96
**Control (4 h)**	22.38	23.85	19.63	36.18	34.06	32.02	34.96	38.24	39.76	37.96	37.96	42.10
**TII (4h)**	224.85	234.17	213.50	236.83	269.97	252.75	578.89	500.57	510.41	551.92	624.61	495.40
**Control (6 h)**	27.88	21.86	20.37	20.45	20.37	22.60	46.32	47.93	43.38	52.65	49.75	61.26
**TII (6 h)**	318.27	321.59	314.30	340.84	ND	348.16	1230.60	1178.61	1092.12	1290.25	1117.32	1174.12
**Control (8 h)**	52.86	24.37	25.61	20.75	26.85	30.21	46.49	43.55	52.15	46.97	48.53	48.65
**TII (8 h)**	352.15	296.43	316.28	347.49	323.58	349.49	2187.18	2224.17	2181.98	2287.14	2839.70	1860.07
**Control (16 h)**	10.41	8.95	9.90	9.27	10.47	11.77	ND	11.23	11.09	12.85	6.33	10.81
**TII (16 h)**	2721.11	2703.31	1900.93	2000.71	1150.10	1084.90	1037.36	929.32	1173.02	985.77	825.87	507.40
**Control (24 h)**	26.05	17.06	16.45	18.50	25.10	24.00	13.77	13.18	15.27	13.45	16.12	19.76
**TII (24 h)**	599.41	588.37	589.84	552.01	713.13	471.51	3888.00	3653.95	3210.97	4027.18	3818.32	3737.74

EXCLI Journal 2023;22:295-309 – ISSN 1611-2156
Received: December 06, 2022, accepted: February 20, 2023, published: February 24, 2023

Supplementary Table 2: Raw data concerning Figure 1B. C20 microglial cells were stimulated with a mixture of proinflammatory cytokines, including TNFα, IL-1β and IFNγ (TII), each used at the same concentration of 50 pg/ml in the cocktail. Cells were treated for different times, namely 4 h, 16 h and 24 h. After the incubation period, the mRNA expression levels of the interleukin-6 (IL-6) were quantified through real-time qPCR using the comparative quantitation method based upon the −ΔΔCt method. Relative mRNA concentrations were calculated from the take-off point of reactions (threshold cycle, Ct), using the expression level of the Control as Calibrator (Cal.) and the expression level of β-actin as normalizing gene. Ct values were obtained at fix threshold of 0.15, in order to combine data from different experiments. Each sample was assayed in triplicate. Raw data presented in the table include Ct values, Ct average and Ct standard deviation (SD), relative quantitation to calibrator (Rel. Quant. to Cal.) and melting temperature (Tm) of the amplified products, for both IL-6 and β-actin mRNA. If Ct SD was > 0.5, one outlier value was excluded from the analysis (please refer to numbers in bold). The last column includes the normalized expression values (Normalized Rel. Quant. to Cal.) for the IL-6 mRNA used to generate Figure 1B and reported in the graph as fold variation versus Control at different times.

**Experiment (number/biological replicates)**	**Treatment**	**Technical Replicates**	**mRNA IL-6**					**mRNA β-actin (Normalizing gene)**					**-ΔΔCt**
			**Ct values**	**Ct Average**	**Ct SD**	**Rel. Quant. to Cal.**	**Tm**	**Ct values**	**Ct Average**	**Ct SD**	**Rel. Quant. to Cal.**	**Tm**	**Normalized Rel. Quant. to Cal.**
1	**Control (4 h)**	1	28.73	28.91	0.20	1.13	79.85	15.07	15.01	0.40	0.96	86.6	1.10
		1	28.88	28.91	0.20	1.02	79.85	15.38	15.01	0.40	0.77	86.6	0.99
		1	29.12	28.91	0.20	0.86	79.83	14.58	15.01	0.40	1.35	86.6	0.84
	**TII (4 h)**	2	24.43	24.89	0.48	22.32	79.83	14.81	15.06	0.22	1.15	86.6	22.86
		2	24.86	24.89	0.48	16.56	79.83	15.11	15.06	0.22	0.93	87.1	16.97
		2	25.39	24.89	0.48	11.47	79.85	15.25	15.06	0.22	0.85	87.1	11.75
2	**Control (4 h)**	3	29.70	29.30	0.35	0.76	79.83	**17.24**				86.3	
		3	29.06	29.30	0.35	1.18	79.85	15.50	15.30	0.28	0.87	86.3	1.17
		3	29.15	29.30	0.35	1.11	79.83	15.10	15.30	0.28	1.15	86.8	1.10
	**TII (4 h)**	4	23.11	23.12	0.06	73.18	79.70	15.36	15.54	0.43	0.96	87.2	84.05
		4	23.18	23.12	0.06	69.71	80.25	16.03	15.54	0.43	0.60	87.15	80.07
		4	23.06	23.12	0.06	75.76	80.20	15.23	15.54	0.43	1.05	87.15	87.02
3	**Control (4 h)**	5	28.39	28.29	0.11	0.94	79.83	17.18	17.13	0.08	0.97	86.83	0.93
		5	28.31	28.29	0.11	0.99	79.83	17.18	17.13	0.08	0.97	86.8	0.99
		5	28.18	28.29	0.11	1.08	79.83	17.03	17.13	0.08	1.07	86.8	1.08
	**TII (4 h)**	6	26.33	26.28	0.09	3.90	79.70	17.95	17.83	0.24	0.57	86.8	6.26
		6	26.17	26.28	0.09	4.36	80.25	17.55	17.83	0.24	0.75	86.75	6.99
		6	26.34	26.28	0.09	3.87	80.20	17.98	17.83	0.24	0.55	87.2	6.22
4	**Control (4 h)**	7	26.84	26.65	0.18	0.88	79.60	15.91	15.63	0.27	0.82	86.60	0.87
		7	26.64	26.65	0.18	1.01	80.10	15.60	15.63	0.27	1.02	86.60	1.00
		7	26.47	26.65	0.18	1.13	80.10	15.38	15.63	0.27	1.19	86.60	1.12
	**TII (4 h)**	8	22.47	22.39	0.07	18.13	80.10	14.67	14.79	0.28	1.95	87.10	10.00
		8	22.38	22.39	0.07	19.29	80.10	14.59	14.79	0.28	2.06	87.10	10.65
		8	22.32	22.39	0.07	20.11	80.10	15.11	14.79	0.28	1.43	87.10	11.10

EXCLI Journal 2023;22:295-309 – ISSN 1611-2156
Received: December 06, 2022, accepted: February 20, 2023, published: February 24, 2023

Supplementary Table 2: Raw data concerning Figure 1B (cont.)

Experiment (number/biological replicates)	Treatment	Technical Replicates	mRNA IL-6					mRNA β-actin (Normalizing gene)					-ΔΔCt
			Ct values	Ct Average	Ct SD	Rel. Quant. to Cal.	Tm	Ct values	Ct Average	Ct SD	Rel. Quant. to Cal.	Tm	Normalized Rel. Quant. to Cal.
1	Control (16 h)	1	27.69	27.70	0.05	1.01	79.85	15.51	15.45	0.05	0.96	86.6	1.01
		1	27.66	27.70	0.05	1.03	79.85	15.41	15.45	0.05	1.03	86.6	1.03
		1	27.76	27.70	0.05	0.96	79.85	15.44	15.45	0.05	1.01	86.6	0.96
	TII (16 h)	2	22.71	22.69	0.03	31.85	79.85	17.30	17.16	0.23	0.28	86.6	102.82
		2	22.66	22.69	0.03	32.98	79.83	16.89	17.16	0.23	0.37	86.6	106.45
		2	22.71	22.69	0.03	31.85	79.83	17.28	17.16	0.23	0.28	86.6	102.82
2	Control (16 h)	3	27.95	27.71	0.21	0.84	80.35	14.93	15.05	0.19	1.08	86.83	0.84
		3	27.57	27.71	0.21	1.10	79.83	14.94	15.05	0.19	1.08	87.3	1.09
		3	27.60	27.71	0.21	1.08	79.83	15.27	15.05	0.19	0.86	87.3	1.07
	TII (16 h)	4	22.69	22.66	0.21	32.37	79.85	15.98	16.05	0.14	0.52	87.25	64.70
		4	22.85	22.66	0.21	28.97	79.83	16.21	16.05	0.14	0.45	87.3	57.91
		4	22.43	22.66	0.21	38.76	79.70	15.96	16.05	0.14	0.53	87.2	77.47
3	Control (16 h)	5	28.20	27.95	0.22	0.84	80.33	16.44	16.43	0.13	1.00	86.85	0.84
		5	27.79	27.95	0.22	1.11	80.33	16.56	16.43	0.13	0.92	86.83	1.11
		5	27.85	27.95	0.22	1.07	79.83	16.30	16.43	0.13	1.10	86.83	1.07
	TII (16 h)	6	25.31	25.28	0.10	6.22	79.83	16.69	16.71	0.12	0.84	86.8	7.50
		6	25.36	25.28	0.10	6.01	79.83	16.60	16.71	0.12	0.89	86.8	7.24
		6	25.17	25.28	0.10	6.85	80.25	16.83	16.71	0.12	0.76	86.8	8.26
4	Control (16 h)	7	29.77	29.69	0.24	0.95	80.35	16.44	16.80	0.38	1.28	86.85	0.93
		7	29.89	29.69	0.24	0.87	79.83	16.75	16.80	0.38	1.03	86.8	0.85
		7	29.42	29.69	0.24	1.21	79.83	17.20	16.80	0.38	0.76	86.8	1.18
	TII (16 h)	8	22.93	23.15	0.20	108.63	80.33	16.76	16.70	0.05	1.03	86.83	101.55
		8	23.21	23.15	0.20	89.47	80.33	16.67	16.70	0.05	1.09	86.8	83.64
		8	23.32	23.15	0.20	82.90	80.33	16.67	16.70	0.05	1.09	86.8	77.50

EXCLI Journal 2023;22:295-309 – ISSN 1611-2156
Received: December 06, 2022, accepted: February 20, 2023, published: February 24, 2023

Supplementary Table 2: Raw data concerning Figure 1B (cont.)

Experiment (number/biological replicates)	Treatment	Technical Replicates	mRNA IL-6					mRNA β-actin (Normalizing gene)					-ΔΔCt
			Ct values	Ct Average	Ct SD	Rel. Quant. to Cal.	Tm	Ct values	Ct Average	Ct SD	Rel. Quant. to Cal.	Tm	Normalized Rel. Quant. to Cal.
**1**	**Control (24 h)**	1	28.07	27.93	0.14	0.91	79.85	18.53	18.70	0.30	1.13	86.6	0.90
		1	27.79	27.93	0.14	1.10	80.33	18.52	18.70	0.30	1.13	87.1	1.09
		1	27.94	27.93	0.14	1.00	80.33	19.05	18.70	0.30	0.78	87.1	0.98
	**TII (24 h)**	2	21.28	21.16	0.12	100.66	79.85	20.16	20.00	0.15	0.36	86.6	247.47
		2	21.14	21.16	0.12	110.92	79.85	19.85	20.00	0.15	0.45	86.6	272.69
		2	21.05	21.16	0.12	118.06	79.85	20.00	20.00	0.15	0.41	86.6	290.24
**2**	**Control (24 h)**	3	26.78	26.49	0.26	0.82	80.35	16.68	16.51	0.19	0.89	87.35	0.81
		3	26.40	26.49	0.26	1.06	80.25	16.53	16.51	0.19	0.98	87.7	1.06
		3	26.29	26.49	0.26	1.15	80.20	16.31	16.51	0.19	1.15	87.65	1.14
	**TII (24 h)**	4	21.31	21.05	0.23	36.25	80.33	16.79	16.83	0.11	0.82	87.3	45.28
		4	20.97	21.05	0.23	45.89	79.83	16.75	16.83	0.11	0.84	87.3	57.31
		4	20.86	21.05	0.23	49.52	79.85	16.95	16.83	0.11	0.74	87.25	61.85
**3**	**Control (24 h)**	5	25.89	25.89	0.08	1.00	80.33	17.04	17.04	0.05	1.00	86.85	1.00
		5	25.80	25.89	0.08	1.06	80.25	17.09	17.04	0.05	0.97	86.75	1.06
		5	25.97	25.89	0.08	0.94	80.20	16.99	17.04	0.05	1.04	86.7	0.94
	**TII (24 h)**	6	23.32	23.10	0.19	5.92	80.33	16.51	16.34	0.15	1.44	86.83	3.64
		6	22.99	23.10	0.19	7.45	79.83	16.24	16.34	0.15	1.74	86.83	4.58
		6	22.98	23.10	0.19	7.50	79.83	16.28	16.34	0.15	1.69	86.8	4.61
**4**	**Control (24 h)**	7	28.26	28.17	0.09	0.94	80.15	15.63	15.75	0.35	1.09	86.65	0.92
		7	28.07	28.17	0.09	1.07	80.15	16.15	15.75	0.35	0.76	86.70	1.05
		7	28.18	28.17	0.09	0.99	80.10	15.47	15.75	0.35	1.21	86.60	0.97
	**TII (24 h)**	8	23.28	23.09	0.32	29.65	79.60	16.39	16.03	0.36	0.64	86.60	35.58
		8	23.26	23.09	0.32	30.06	79.60	16.07	16.03	0.36	0.80	86.60	36.08
		8	22.72	23.09	0.32	43.71	80.10	15.67	16.03	0.35	1.06	87.10	52.46

EXCLI Journal 2023;22:295-309 – ISSN 1611-2156
Received: December 06, 2022, accepted: February 20, 2023, published: February 24, 2023

Supplementary Table 3: Raw data concerning Figure 2A and Table 1. C20 microglial cells were stimulated with a mixture of proinflammatory cytokines, including TNFα, IL-1β and IFNγ (TII), each used at the same concentration of 50 pg/ml in the cocktail. Cells were treated for different times, from 2 h up to 24 h. After the incubation period, the concentration of the monocyte chemoattractant protein-1 (MCP-1, pg/ml) in the incubation medium was measured by a specific ELISA assay. The amounts of MCP-1 measured in the incubation medium after different times of incubation are reported in this table. Data are shown as pg/ml and were obtained from 3 independent experiments, each including 4 biological replicates per treatment. ND = not detectable. The ND values were excluded from the analysis.

**Treatments**	**Experiment n. 1**				**Experiment n. 2**				**Experiment n. 3**			
	**MCP-1 Concentration (pg/ml)**				**MCP-1 Concentration (pg/ml)**				**MCP-1 Concentration (pg/ml)**			
**Control (2 h)**	155.37	49.18	53.14	24.69	39.10	43.52	54.88	33.19	ND	22.02	84.02	27.70
**TII (2 h)**	205.74	313.83	647.02	174.11	305.70	286.63	361.06	344.75	26.45	105.72	69.09	479.08
**Control (4 h)**	108.44	54.95	212.24	ND	79.83	114.83	119.70	77.83	64.13	43.33	150.49	22.64
**TII (4 h)**	974.29	1200.50	1064.04	1183.21	1866.78	2119.12	1767.16	1786.42	1715.87	658.92	2175.26	2664.79
**Control (6 h)**	134.84	42.48	131.48	123.31	215.89	207.13	214.86	236.88	175.91	153.28	126.41	156.54
**TII (6 h)**	2033.39	1055.43	803.12	1042.91	4789.24	4529.09	4652.77	4049.14	3304.37	3658.35	3606.57	4708.59
**Control (8 h)**	198.03	194.10	276.82	222.53	226.32	120.59	241.13	183.90	217.53	174.00	266.14	232.66
**TII (8 h)**	2511.00	2401.00	2570.95	2140.24	4962.82	4383.02	4902.78	5549.02	4934.02	5340.81	5206.32	4725.01
**Control (16 h)**	149.44	117.27	109.40	110.63	195.93	140.16	151.87	147.62	102.40	111.44	90.61	92.28
**TII (16 h)**	3779.85	2938.84	2790.47	3531.46	5072.27	7412.37	6045.04	6157.21	5230.03	5438.10	6036.75	6989.28
**Control (24 h)**	290.51	183.77	183.41	179.90	226.32	264.89	276.98	282.52	161.22	268.23	268.23	279.27
**TII (24 h)**	8225.66	7667.60	6186.11	4992.82	9768.53	10236.92	10552.39	8453.45	10527.84	12792.65	13155.67	13085.69

EXCLI Journal 2023;22:295-309 – ISSN 1611-2156
Received: December 06, 2022, accepted: February 20, 2023, published: February 24, 2023

Supplementary Table 4: Raw data concerning Figure 2B. C20 microglial cells were stimulated with with a mixture of proinflammatory cytokines, including TNFα, IL-1β and IFNγ (TII), each used at the same concentration of 50 pg/ml in the cocktail. Cells were treated for different times, namely 4 h, 16 h and 24 h. After the incubation period, the mRNA expression levels of the monocyte chemoattractant protein-1 (MCP-1) were quantified through real-time qPCR using the comparative quantitation method based upon the −ΔΔCt method. Relative mRNA concentrations were calculated from the take-off point of reactions (threshold cycle, Ct), using the expression level of the Control as Calibrator (Cal.) and the expression level of β-actin as normalizing gene. Ct values were obtained at fix threshold of 0.15, in order to combine data from different experiments. Each sample was assayed in triplicate. Raw data presented in the table include Ct values, Ct average and Ct standard deviation (SD), relative quantitation to calibrator (Rel. Quant. to Cal.) and melting temperature (Tm) of the amplified products, for both MCP-1 and β-actin mRNA. If Ct SD was > 0.5, one outlier value was excluded from the analysis (please refer to numbers in bold). The last column includes the normalized expression values (Normalized Rel. Quant. to Cal.) for the MCP-1 mRNA used to generate Fig. 2B and reported in the graph as fold variation versus Control at different times.

**Experiment (number/ biological replicates)**	**Treatment**	**Technical Replicates**	**mRNA MCP-1**					**mRNA β-actin (Normalizing gene)**					**-ΔΔCt**
			**Ct values**	**Ct Average**	**Ct SD**	**Rel. Quant. to Cal.**	**Tm**	**Ct values**	**Ct Average**	**Ct SD**	**Rel. Quant. to Cal.**	**Tm**	**Normalized Rel. Quant. to Cal.**
**1**	**Control (4 h)**	1	25.17	24.90	0.23	0.83	83.25	15.07	15.01	0.40	0.96	86.6	0.81
		1	24.76	24.90	0.23	1.10	83.20	15.38	15.01	0.40	0.77	86.6	1.07
		1	24.76	24.90	0.23	1.10	83.20	14.58	15.01	0.40	1.35	86.6	1.07
	**TII (4 h)**	2	18.71	18.35	0.32	72.84	83.15	14.81	15.06	0.22	1.15	86.6	74.62
		2	18.11	18.35	0.32	110.41	83.65	15.11	15.06	0.22	0.93	87.1	113.10
		2	18.23	18.35	0.32	101.59	83.65	15.25	15.06	0.22	0.85	87.1	104.07
**2**	**Control (4 h)**	3	25.83	25.62	0.26	0.86	83.33	**17.24**				86.3	
		3	25.33	25.62	0.26	1.22	83.33	15.50	15.30	0.28	0.87	86.3	1.21
		3	25.70	25.62	0.26	0.95	83.33	15.10	15.30	0.28	1.15	86.8	0.94
	**TII (4 h)**	4	19.14	19.16	0.11	89.26	83.30	15.36	15.54	0.43	0.96	87.2	102.53
		4	19.28	19.16	0.11	81.01	83.30	16.03	15.54	0.43	0.60	87.15	93.05
		4	19.06	19.16	0.11	94.35	83.30	15.23	15.54	0.43	1.05	87.15	108.37
**3**	**Control (4 h)**	5	27.70	27.76	0.05	1.04	83.30	17.18	17.13	0.08	0.97	86.83	1.04
		5	27.78	27.76	0.05	0.99	83.33	17.18	17.13	0.08	0.97	86.8	0.99
		5	27.80	27.76	0.05	0.97	83.33	17.03	17.13	0.08	1.07	86.8	0.97
	**TII (4 h)**	6	23.79	23.76	0.03	15.67	83.25	17.95	17.83	0.24	0.57	86.8	25.16
		6	23.76	23.76	0.03	16.00	83.25	17.55	17.83	0.24	0.75	86.75	25.69
		6	23.73	23.76	0.03	16.34	83.25	17.98	17.83	0.24	0.55	87.2	26.23
**4**	**Control (4h)**	7	22.42	22.73	0.44	1.24	83.10	15.91	15.63	0.27	0.82	86.6	1.23
		7	23.04	22.73	0.44	0.81	83.10	15.60	15.63	0.27	1.02	86.6	0.80
		7	**No Ct**				83.10	15.38	15.63	0.27	1.19	86.6	
	**TII (4h)**	8	17.65	17.40	0.36	33.82	83.60	14.67	14.79	0.28	1.95	87.1	18.67
		8	17.14	17.40	0.36	48.17	83.10	14.59	14.79	0.28	2.06	87.1	26.59
		8	**28.30**				83.10	15.11	14.79	0.28	1.43	87.1	

EXCLI Journal 2023;22:295-309 – ISSN 1611-2156
Received: December 06, 2022, accepted: February 20, 2023, published: February 24, 2023

Supplementary Table 4: Raw data concerning Figure 2B (cont.)

Experiment (number/ biological replicates)	Treatment	Technical Replicates	mRNA MCP-1					mRNA β-actin (Normalizing gene)					-ΔΔCt
			Ct values	Ct Average	Ct SD	Rel. Quant. to Cal.	Tm	Ct values	Ct Average	Ct SD	Rel. Quant. to Cal.	Tm	Normalized Rel. Quant. to Cal.
1	Control (16 h)	1	23.12	23.14	0.08	1.01	83.83	15.51	15.45	0.05	0.96	86.6	1.01
		1	23.23	23.14	0.08	0.94	83.80	15.41	15.45	0.05	1.03	86.6	0.94
		1	23.07	23.14	0.08	1.05	83.25	15.44	15.45	0.05	1.01	86.6	1.05
	TII (16 h)	2	17.50	17.49	0.02	49.87	83.75	17.30	17.16	0.23	0.28	86.6	160.97
		2	17.47	17.49	0.02	50.91	83.70	16.89	17.16	0.23	0.37	86.6	164.35
		2	17.51	17.49	0.02	49.52	83.65	17.28	17.16	0.23	0.28	86.6	159.86
2	Control (16 h)	3	24.36	24.38	0.13	1.01	83.35	14.93	15.05	0.19	1.08	86.83	1.01
		3	24.52	24.38	0.13	0.91	83.33	14.94	15.05	0.19	1.08	87.3	0.90
		3	24.26	24.38	0.13	1.09	83.33	15.27	15.05	0.19	0.86	87.3	1.08
	TII (16 h)	4	18.41	18.29	0.12	62.68	83.33	15.98	16.05	0.14	0.52	87.25	125.28
		4	18.16	18.29	0.12	74.54	83.33	16.21	16.05	0.14	0.45	87.3	148.98
		4	18.29	18.29	0.12	68.12	83.30	15.96	16.05	0.14	0.53	87.2	136.14
3	Control (16 h)	5	25.99	25.96	0.11	0.98	83.35	16.44	16.43	0.13	1.00	86.85	0.98
		5	25.84	25.96	0.11	1.09	83.33	16.56	16.43	0.13	0.92	86.83	1.08
		5	26.05	25.96	0.11	0.94	83.30	16.30	16.43	0.13	1.10	86.83	0.94
	TII (16 h)	6	21.52	21.51	0.11	21.71	83.33	16.69	16.71	0.12	0.84	86.8	26.18
		6	21.40	21.51	0.11	23.59	83.30	16.60	16.71	0.12	0.89	86.8	28.45
		6	21.62	21.51	0.11	20.25	83.25	16.83	16.71	0.12	0.76	86.8	24.42
4	Control (16 h)	7	25.19	25.23	0.08	1.03	83.25	16.44	16.80	0.38	1.28	86.85	1.00
		7	25.18	25.23	0.08	1.04	83.25	16.75	16.80	0.38	1.03	86.8	1.01
		7	25.32	25.23	0.08	0.94	83.25	17.20	16.80	0.38	0.76	86.8	0.92
	TII (16 h)	8	18.10	18.14	0.22	140.07	83.85	16.76	16.70	0.05	1.03	86.83	130.94
		8	17.94	18.14	0.22	156.50	83.85	16.67	16.70	0.05	1.09	86.8	146.29
		8	18.37	18.14	0.22	116.16	83.25	16.67	16.70	0.05	1.09	86.8	108.59

EXCLI Journal 2023;22:295-309 – ISSN 1611-2156
Received: December 06, 2022, accepted: February 20, 2023, published: February 24, 2023

Supplementary Table 4: Raw data concerning Figure 2B (cont.)

Experiment (number/ biological replicates)	Treatment	Technical Replicates	mRNA MCP-1					mRNA β-actin (Normalizing gene)					-ΔΔCt
			Ct values	Ct Average	Ct SD	Rel. Quant. to Cal.	Tm	Ct values	Ct Average	Ct SD	Rel. Quant. to Cal.	Tm	Normalized Rel. Quant. to Cal.
1	Control (24 h)	1	23.76	23.80	0.07	1.03	83.83	18.53	18.70	0.30	1.13	86.6	1.01
		1	23.88	23.80	0.07	0.94	83.65	18.52	18.70	0.30	1.13	87.1	0.93
		1	23.75	23.80	0.07	1.03	83.65	19.05	18.70	0.30	0.78	87.1	1.02
	TII (24 h)	2	19.54	19.58	0.15	19.12	83.80	20.16	20.00	0.15	0.36	86.6	47.00
		2	19.75	19.58	0.15	16.53	83.75	19.85	20.00	0.15	0.45	86.6	40.63
		2	19.46	19.58	0.15	20.21	83.20	20.00	20.00	0.15	0.41	86.6	49.68
2	Control (24 h)	3	24.19	24.20	0.08	1.01	83.85	16.68	16.51	0.19	0.89	87.35	1.00
		3	24.29	24.20	0.08	0.94	83.30	16.53	16.51	0.19	0.98	87.7	0.94
		3	24.13	24.20	0.08	1.05	83.80	16.31	16.51	0.19	1.15	87.65	1.05
	TII (24 h)	4	19.27	19.15	0.10	30.55	83.33	16.79	16.83	0.11	0.82	87.3	38.16
		4	19.13	19.15	0.10	33.67	83.33	16.75	16.83	0.11	0.84	87.3	42.05
		4	19.06	19.15	0.10	35.34	83.33	16.95	16.83	0.11	0.74	87.25	44.14
3	Control (24 h)	5	26.41	26.43	0.13	1.01	83.85	17.04	17.04	0.05	1.00	86.85	1.01
		5	26.56	26.43	0.13	0.91	83.25	17.09	17.04	0.05	0.97	86.75	0.91
		5	26.31	26.43	0.13	1.08	83.25	16.99	17.04	0.05	1.04	86.7	1.08
	TII (24 h)	6	20.73	20.66	0.10	51.86	83.80	16.51	16.34	0.15	1.44	86.83	31.89
		6	20.70	20.66	0.10	52.95	83.30	16.24	16.34	0.15	1.74	86.83	32.56
		6	20.54	20.66	0.10	59.16	83.33	16.28	16.34	0.15	1.69	86.8	36.38
4	Control (24 h)	7	24.71	24.35	0.38	0.78	83.65	15.63	15.75	0.36	1.09	86.65	0.76
		7	23.96	24.35	0.38	1.31	83.65	16.15	15.75	0.36	0.76	86.70	1.28
		7	24.37	24.35	0.38	0.98	83.15	15.47	15.75	0.36	1.21	86.60	0.97
	TII (24 h)	8	19.18	19.14	0.06	35.92	83.10	16.39	16.04	0.36	0.64	86.60	43.11
		8	19.16	19.14	0.06	36.42	83.10	16.07	16.04	0.36	0.80	86.60	43.71
		8	19.07	19.14	0.06	38.76	83.10	15.67	16.04	0.36	1.06	87.10	46.52

EXCLI Journal 2023;22:295-309 – ISSN 1611-2156
Received: December 06, 2022, accepted: February 20, 2023, published: February 24, 2023

Supplementary Table 5: Raw data concerning Figure 2C. C20 microglial cells were stimulated with a mixture of proinflammatory cytokines, including TNFα, IL-1β and IFNγ (TII), each used at the same concentration of 50 pg/ml in the cocktail. Cells were treated for different times, from 2 h up to 24 h. After the incubation period, the concentration of interleukin 8 (IL-8, pg/ml) in the incubation medium was measured by a specific ELISA assay. The amounts of IL-8 measured in the incubation medium after different times of incubation are reported in this table. Data are shown as pg/ml and were obtained from 4 independent experiments, each including 4 biological replicates per treatment. ND = not detectable. The ND values were excluded from the analysis.

**Treatments**	**Experiment n. 1**				**Experiment n. 2**				**Experiment n. 3**				**Experiment n. 4**			
	**IL-8 Concentration (pg/ml)**				**IL-8 Concentration (pg/ml)**				**IL-8 Concentration (pg/ml)**				**IL-8 Concentration (pg/ml)**			
**Control (2 h)**	7.39	5.85	5.96	5.05	16.54	15.92	16.14	16.69	11.82	11.82	12.81	10.35	11.36	12.33	11.45	10.61
**TII (2 h)**	58.68	76.36	74.23	87.66	82.86	99.68	87.71	91.70	64.37	72.92	84.52	83.17	77.15	73.17	84.25	80.50
**Control (4 h)**	11.11	10.09	10.06	9.66	17.56	16.71	17.75	17.22	13.17	15.46	11.82	15.05	16.97	17.20	12.50	15.67
**TII (4 h)**	2371.80	1852.86	1730.25	1984.92	357.05	359.76	408.47	391.13	564.62	377.39	645.86	678.27	1242.22	920.95	1877.53	1733.37
**Control (6 h)**	21.15	13.65	12.52	12.85	18.01	19.10	19.02	18.06	13.64	13.08	17.20	12.42	23.27	18.00	15.05	16.04
**TII (6 h)**	1801.25	1294.81	1759.89	2541.08	1008.43	777.30	860.00	876.81	1658.97	735.18	771.12	3820.19	1151.77	1812.76	1019.58	ND
**Control (8 h)**	23.60	15.49	13.15	20.19	18.36	17.48	19.10	18.58	14.31	15.21	18.60	15.88	24.52	17.14	34.82	23.41
**TII (8 h)**	2712.21	2096.94	1942.62	2290.50	1098.31	984.75	1046.62	1140.65	4280.23	5289.99	2932.39	1342.23	1503.25	4367.09	5940.35	10244.15
**Control (16 h)**	12.67	9.66	8.71	7.53	17.88	17.38	18.04	18.22	16.69	21.26	26.53	18.42	29.44	15.83	17.88	19.51
**TII (16 h)**	3665.34	3332.44	4388.97	6756.02	1064.73	1039.81	1251.35	1232.87	1807.31	1730.38	1807.31	1837.96	5781.34	4063.77	6928.46	6095.61
**Control (24 h)**	16.88	14.40	9.89	11.11	18.63	18.69	17.72	17.25	26.13	17.94	24.52	101.28	25.51	19.51	21.53	20.53
**TII (24 h)**	9051.22	8463.53	6186.25	5944.69	1776.25	1812.46	1758.45	1716.94	9866.09	4130.58	2804.56	3086.85	9005.83	3868.52	10328.83	13246.23

EXCLI Journal 2023;22:295-309 – ISSN 1611-2156
Received: December 06, 2022, accepted: February 20, 2023, published: February 24, 2023

Supplementary Table 6: Raw data concerning Figure 2D. C20 microglial cells were stimulated with with a mixture of proinflammatory cytokines, including TNFα, IL-1β and IFNγ (TII), each used at the same concentration of 50 pg/ml in the cocktail. Cells were treated for different times, namely 4 h, 16 h and 24 h. After the incubation period, the mRNA expression levels of the monocyte chemoattractant protein-1 (MCP-1) were quantified through real-time qPCR using the comparative quantitation method based upon the −ΔΔCt method. Relative mRNA concentrations were calculated from the take-off point of reactions (threshold cycle, Ct), using the expression level of the Control as Calibrator (Cal.) and the expression level of β-actin as normalizing gene. Ct values were obtained at fix threshold of 0.15, in order to combine data from different experiments. Each sample was assayed in triplicate. Raw data presented in the table include Ct values, Ct average and Ct standard deviation (SD), relative quantitation to calibrator (Rel. Quant. to Cal.) and melting temperature (Tm) of the amplified products, for both MCP-1 and β-actin mRNA. If Ct SD was > 0.5, one outlier value was excluded from the analysis (please refer to numbers in bold). Values for Control and TII at 4 h derived from Experiment 4 were excluded from the analysis since SD remained > 0.5 despite the exclusion of 1 outlier (last column, values in bold). The last column includes the normalized expression values (Normalized Rel. Quant. to Cal.) for the IL-8 mRNA used to generate Figure 2D and reported in the graph as fold variation versus Control at different times.

Experiment (number/biological replicates)	Treatment	Technical Replicates	mRNA IL-8					mRNA β-actin (Normalizing gene)					-ΔΔCt
			Ct values	Ct Average	Ct SD	Rel. Quant. to Cal.	Tm	Ct values	Ct Average	Ct SD	Rel. Quant. to Cal.	Tm	Normalized Rel. Quant. to Cal.
1	Control (4 h)	1	**No Ct**				81.3	15.07	15.01	0.40	0.96	86.6	
		1	29.57	29.13	0.62	0.74	81.75	15.38	15.01	0.40	0.77	86.6	0.72
		1	28.69	29.13	0.62	1.36	81.75	14.58	15.01	0.40	1.35	86.6	1.32
	TII (4 h)	2	19.10	19.17	0.09	1045.52	81.6	14.81	15.06	0.22	1.15	86.6	1071.05
		2	**20.61**				81.6	15.11	15.06	0.22	0.93	87.1	
		2	19.23	19.17	0.09	955.43	82.1	15.25	15.06	0.22	0.85	87.1	978.76
2	Control (4 h)	3	29.34	29.24	0.22	0.94	81.33	**17.24**				86.3	
		3	29.40	29.24	0.22	0.90	81.8	15.5	15.30	0.28	0.87	86.3	0.89
		3	28.99	29.24	0.22	1.19	81.8	15.1	15.30	0.28	1.15	86.8	1.18
	TII (4 h)	4	18.81	18.93	0.12	1382.76	81.85	15.36	15.54	0.43	0.96	87.2	1588.23
		4	18.92	18.93	0.12	1281.25	81.85	16.03	15.54	0.43	0.60	87.15	1471.64
		4	19.05	18.93	0.12	1170.84	81.83	15.23	15.54	0.43	1.05	87.15	1344.83
3	Control (4 h)	5	29.41	29.61	0.17	1.14	81.85	17.18	17.13	0.08	0.97	86.83	1.14
		5	29.68	29.61	0.17	0.95	81.85	17.18	17.13	0.08	0.97	86.8	0.95
		5	29.72	29.61	0.17	0.92	81.8	17.03	17.13	0.08	1.07	86.8	0.92
	TII (4 h)	6	25.38	25.31	0.14	18.68	81.8	17.95	17.82	0.24	0.57	86.8	29.99
		6	25.14	25.31	0.14	22.06	81.8	17.55	17.82	0.24	0.75	86.75	35.42
		6	25.39	25.31	0.14	18.55	81.83	17.98	17.82	0.24	0.55	87.2	29.78
4	Control (4 h)	7	28.47	27.79	0.97	0.62	81.10	15.91	15.63	0.27	0.82	86.60	**0.62**
		7	**No Ct**				79.60	15.60	15.63	0.27	1.02	86.60	
		7	27.10	27.79	0.97	1.61	81.60	15.38	15.63	0.27	1.19	86.60	**1.59**
	TII (4 h)	8	**28.30**				81.10	14.67	14.79	0.28	1.95	87.10	
		8	22.57	20.43	3.03	37.14	81.60	14.59	14.79	0.28	2.06	87.10	**20.50**
		8	18.28	20.43	3.03	726.59	81.60	15011	14.79	0.28	1.43	87.10	**401.03**

EXCLI Journal 2023;22:295-309 – ISSN 1611-2156
Received: December 06, 2022, accepted: February 20, 2023, published: February 24, 2023

Supplementary Table 6: Raw data concerning Figure 2D (cont.)

Experiment (number/biological replicates)	Treatment	Technical Replicates	mRNA IL-8					mRNA β-actin (Normalizing gene)					-ΔΔCt
			Ct values	Ct Average	Ct SD	Rel. Quant. to Cal.	Tm	Ct values	Ct Average	Ct SD	Rel. Quant. to Cal.	Tm	Normalized Rel. Quant. to Cal.
1	Control (16 h)	1	28.54	28.64	0.09	1.07	81.83	15.51	15.45	0.05	1.03	86.6	1.05
		1	28.72	28.64	0.09	0.95	81.85	15.41	15.45	0.05	1.01	86.6	0.93
		1	28.66	28.64	0.09	0.99	81.83	15.44	15.45	0.05	0.28	86.6	0.97
	TII (16 h)	2	19.47	19.12	0.33	576.03	81.75	17.3	17.16	0.23	0.37	86.6	1859.42
		2	19.04	19.12	0.33	776.05	81.75	16.89	17.16	0.23	0.28	86.6	2505.08
		2	18.83	19.12	0.33	897.64	81.6	17.28	17.16	0.23	1.03	86.6	2897.59
2	Control (16 h)	3	28.19	28.39	0.43	1.15	81.85	14.93	15.05	0.19	1.08	86.83	1.14
		3	28.88	28.39	0.43	0.71	81.3	14.94	15.05	0.19	1.08	87.3	0.71
		3	28.09	28.39	0.43	1.23	81.3	15.27	15.05	0.19	0.86	87.3	1.22
	TII (16 h)	4	19.94	19.84	0.08	348.90	81.85	15.98	16.05	0.14	0.52	87.25	697.29
		4	19.80	19.84	0.08	384.45	81.8	16.21	16.05	0.14	0.45	87.3	768.35
		4	19.79	19.84	0.08	387.13	81.78	15.96	16.05	0.14	0.53	87.2	773.70
3	Control (16 h)	5	29.79	29.30	0.45	0.71	81.85	16.44	16.43	0.13	1.00	86.85	0.71
		5	28.91	29.30	0.45	1.31	81.85	16.56	16.43	0.13	0.92	86.83	1.31
		5	29.20	29.30	0.45	1.07	81.35	16.3	16.43	0.13	1.10	86.83	1.07
	TII (16 h)	6	23.71	23.54	0.17	48.17	81.85	16.69	16.7	0.12	0.84	86.8	58.09
		6	23.37	23.54	0.17	60.97	81.8	16.6	16.7	0.12	0.89	86.8	73.53
		6	23.55	23.54	0.17	53.82	81.8	16.83	16.7	0.12	0.76	86.8	64.90
4	Control (16 h)	7	**32.66**					16.44	16.8	0.38	1.28	86.85	
		7	31.39	31.29	0.15	0.93	81.25	16.75	16.8	0.38	1.03	86.8	0.91
		7	31.18	31.29	0.15	1.08	81.25	17.2	16.8	0.38	0.76	86.8	1.05
	TII (16 h)	8	19.46	19.28	0.28	3628.10	81.83	16.76	16.7	0.05	1.03	86.83	3391.51
		8	18.96	19.28	0.28	5130.91	81.8	16.67	16.7	0.05	1.09	86.8	4796.33
		8	19.43	19.28	0.28	3704.34	81.8	16.67	16.7	0.05	1.09	86.8	3462.78

EXCLI Journal 2023;22:295-309 – ISSN 1611-2156
Received: December 06, 2022, accepted: February 20, 2023, published: February 24, 2023

Supplementary Table 6: Raw data concerning Figure 2D (cont.)

Experiment (number/biological replicates)	Treatment	Technical Replicates	mRNA IL-8					mRNA β-actin (Normalizing gene)					-ΔΔCt
			Ct values	Ct Average	Ct SD	Rel. Quant. to Cal.	Tm	Ct values	Ct Average	Ct SD	Rel. Quant. to Cal.	Tm	Normalized Rel. Quant. to Cal.
1	Control (24 h)	1	28.86	28.85	0.31	0.99	81.83	18.53	18.7	0.30	1.13	86.6	0.98
		1	29.15	28.85	0.31	0.81	81.6	18.52	18.7	0.30	1.13	87.1	0.80
		1	28.53	28.85	0.31	1.25	81.65	19.05	18.7	0.30	0.78	87.1	1.23
	TII (24 h)	2	19.61	19.41	0.2	603.27	81.85	20.16	20	0.15	0.36	86.6	1483.16
		2	19.40	19.41	0.2	697.80	81.83	19.85	20	0.15	0.45	86.6	1715.55
		2	19.21	19.41	0.2	796.02	81.75	20	20	0.15	0.41	86.6	1957.04
2	Control (24 h)	3	27.79	28.17	0.42	1.30	81.85	16.68	16.51	0.19	0.89	87.35	1.30
		3	28.11	28.17	0.42	1.04	81.83	16.53	16.51	0.19	0.98	87.7	1.04
		3	28.62	28.17	0.42	0.73	81.75	16.31	16.51	0.19	1.15	87.65	0.73
	TII (24 h)	4	19.25	19.06	0.17	485.50	81.83	16.79	16.83	0.11	0.82	87.3	606.39
		4	18.97	19.06	0.17	589.49	81.8	16.75	16.83	0.11	0.84	87.3	736.28
		4	18.96	19.06	0.17	593.59	81.85	16.95	16.83	0.11	0.74	87.25	741.40
3	Control (24 h)	5	28.06	28.04	0.03	0.99	81.35	17.04	17.04	0.05	1.00	86.85	0.99
		5	28.05	28.04	0.03	0.99	81.8	17.09	17.04	0.05	0.97	86.75	0.99
		5	28.01	28.04	0.03	1.02	81.83	16.99	17.04	0.05	1.04	86.7	1.02
	TII (24 h)	6	21.24	21.07	0.19	111.43	81.85	16.51	16.34	0.15	1.44	86.83	68.52
		6	20.86	21.07	0.19	145.01	81.85	16.24	16.34	0.15	1.74	86.83	89.17
		6	21.13	21.07	0.19	120.26	81.85	16.28	16.34	0.15	1.69	86.8	73.95
4	Control (24 h)	7	**30.80**				81.70	15.63	15.75	0.36	1.09	86.65	
		7	28.34	28.26	0.11	0.95	81.60	16.15	15.75	0.36	0.76	86.70	0.93
		7	28.18	28.26	0.11	1.06	81.60	15.47	15.75	0.36	1.21	86.60	1.04
	TII (24 h)	8	20.50	20.60	0.11	216.77	81.60	16.39	16.04	0.36	0.64	86.60	260.14
		8	20.60	20.60	0.11	202.25	81.60	16.07	16.04	0.36	0.80	86.60	242.72
		8	20.71	20.60	0.11	187.40	81.60	15.67	16.04	0.36	1.06	87.10	224.90

EXCLI Journal 2023;22:295-309 – ISSN 1611-2156
Received: December 06, 2022, accepted: February 20, 2023, published: February 24, 2023

Supplementary Table 7: Raw data concerning Figure 3A. C20 cells were treated with different concentrations of remifentanil (RF) alone or in combination with TII for 6 h. TII consists in a mixture of proinflammatory cytokines, including TNFα, IL-1β and IFNγ, each used at 50 pg/ml. After the incubation period, cell viability was assed using the MTS assay. Raw data are expressed as Net Optical Density (OD), calculated by subtracting the absorbance values measured at 655 nm to the absorbance values measured at 490 nm (Net OD = OD_{490} – OD_{655}). Two readings were performed, at time 0 (soon after the addition of the MTS solutions, t=0) and after 3 h of incubation at 37 °C in a humidified atmosphere containing 5 % CO_2. Each reading is corrected by the average Net OD measured for the Blanks, i.e. plain medium, not exposed to the cells and incubated for 3 h in the same conditions of the experimental samples. These data are then normalized as percentage of controls. In the table, we reported the Net OD values: [(Net OD at 3 h - Average Net OD for the Blanks at 3h) - (Net OD at t=0 - Average Net OD for the Blanks at t=0)]. These values were used to calculate the normalized data, shown in the table as Normalized Values. The latter were used to generate Figure 3A. RF=remifentanil; TII= mixture of TNFα, IL-1β and IFNγ; Net OD= Net Optical Density

**Treatments**	**Experiment n. 1**					**Experiment n. 2**					**Experiment n. 3**			
	**Net OD**					**Net OD**					**Net OD**			
**Control**	0.476	0.507	0.544	0.311	0.444	0.355	0.362	0.371	0.427	0.364	1.120	1.167	1.194	1.111
**1.25 ng/ml RF**	0.383	0.516	0.437	0.570	0.800	0.376	0.428	0.378	0.442	0.360	1.088	0.990	1.003	0.934
**2.5 ng/ml RF**	0.393	0.544	0.561	0.778	0.574	0.389	0.42	0.432	0.394	0.383	0.946	1.096	0.985	1.114
**5 ng/ml RF**	0.503	0.526	0.507	0.631	0.625	0.356	0.406	0.449	0.403	0.423	0.884	0.837	0.823	1.117
**10 ng/ml RF**	0.328	0.410	0.521	0.685	0.705	0.351	0.372	0.36	0.358	0.331	0.862	0.830	0.877	0.950
**20 ng/ml RF**	0.270	0.382	0.434	0.355	0.421	0.356	0.33	0.362	0.34	0.352	0.865	0.840	0.871	0.873
**TII**	0.634	0.583	0.407	0.583	0.608	0.366	0.406	0.383	0.375	0.394	0.815	0.787	0.764	0.767
**TII + 1.25 ng/ml RF**	0.773	0.610	0.633	0.618	0.605	0.456	0.399	0.422	0.388	0.434	0.844	0.806	0.790	0.765
**TII + 2.5 ng/ml RF**	0.764	0.591	0.632	0.656	0.524	0.403	0.414	0.387	0.427	0.402	0.932	0.827	0.931	0.782
**TII + 5 ng/ml RF**	0.657	0.607	0.655	0.612	0.573	0.431	0.438	0.44	0.45	0.427	0.862	0.992	1.010	0.834
**TII + 10 ng/ml RF**	0.333	0.599	0.616	0.597	0.393	0.393	0.4	0.436	0.422	0.416	1.151	1.167	1.175	1.214
**TII + 20 ng/ml RF**	0.39	0.439	0.594	0.559	0.451	0.336	0.393	0.422	0.355	0.384	1.157	1.082	1.012	0.960

Supplementary Table 7: Raw data concerning Figure 3A (cont.)

Treatments	Experiment n. 1					Experiment n. 2					Experiment n. 3			
	Normalized values					Normalized values					Normalized values			
**Control**	104.29	111.09	119.19	68.14	97.28	94.47	96.33	98.72	113.62	96.86	97.56	101.65	104.01	96.78
**1.25 ng/ml RF**	83.92	113.06	95.75	124.89	175.28	100.05	113.89	100.59	117.62	95.80	94.78	86.24	87.37	81.36
**2.5 ng/ml RF**	86.11	119.19	122.92	170.46	125.76	103.51	111.76	114.95	104.84	101.92	82.41	95.47	85.81	97.04
**5 ng/ml RF**	110.21	115.25	111.09	138.25	136.94	94.73	108.04	119.48	107.24	112.56	77.01	72.92	71.70	97.30
**10 ng/ml RF**	71.87	89.83	114.15	150.08	154.47	93.40	98.99	95.80	95.26	88.08	75.09	72.30	76.40	82.76
**20 ng/ml RF**	59.16	83.70	95.09	77.78	92.24	94.73	87.81	96.33	90.47	93.67	75.36	73.18	75.88	76.05
**TII**	138.91	127.74	89.18	127.74	133.21	97.39	108.04	101.92	99.79	104.84	71.00	68.56	66.56	66.82
**TII + 1.25 ng/ml RF**	169.37	133.65	138.69	135.40	132.56	121.34	106.17	112.29	103.25	115.49	73.53	70.22	68.82	66.65
**TII + 2.5 ng/ml RF**	167.39	129.49	138.47	143.73	114.81	107.24	110.16	102.98	113.62	106.97	81.19	72.05	81.10	68.13
**TII + 5 ng/ml RF**	143.95	132.99	143.51	134.09	125.55	114.69	116.55	117.08	119.74	113.62	75.09	86.42	87.98	72.66
**TII + 10 ng/ml RF**	72.96	131.24	134.97	130.80	86.11	104.58	106.44	116.02	112.29	110.70	100.26	101.65	102.35	105.75
**TII + 20 ng/ml RF**	85.45	96.19	130.15	122.48	98.82	89.41	104.58	112.29	94.47	102.18	100.78	94.25	88.16	83.63

EXCLI Journal 2023;22:295-309 – ISSN 1611-2156
Received: December 06, 2022, accepted: February 20, 2023, published: February 24, 2023

Supplementary Table 8: Raw data concerning Figure 3B. C20 cells were treated with RF alone at different concentration and in combination with TII for 16 h. TII consists in a mixture of proinflammatory cytokines, including TNFα, IL-1β and IFNγ, each used at the same concentration (50 pg/ml). After the incubation period, cell viability was assessed using the MTS assay. Raw data are expressed as Net Optical Density (OD), calculated by subtracting the absorbance values measured at 655 nm to the absorbance values measured at 490 nm (Net OD = OD_{490} – OD_{655}). Two readings were performed, at time 0 (soon after the addition of the MTS solutions, t=0) and after 3 h of incubation at 37 °C in a humidified atmosphere containing 5 % CO_2. Each reading is corrected by the average Net OD measured for the Blanks, i.e. plain medium, not exposed to the cells and incubated for 3 h in the same conditions of the experimental samples. These data are then normalized as percentage of controls. In the table, we reported the Net OD values: [(Net OD at 3 h – Average Net OD for the Blanks at 3 h) - (Net OD at t=0 – Average Net OD for the Blanks at t=0)]. These values were used to calculate the normalized data, shown in the table as Normalized Values. The latter were used to generate Figure 3B. RF=remifentanil; TII= mixture of TNFα, IL-1β and IFNγ; Net OD= Net Optical Density

**Treatments**	**Experiment n. 1**					**Experiment n. 2**					**Experiment n. 3**			
	**Net OD 490 nm**					**Net OD 490 nm**					**Net OD 490 nm**			
**Control**	1.022	1.126	1.095	1.115	0.949	0.764	0.634	0.644	0.565	0.603	1.244	1.154	1.367	1.336
**1.25 ng/ml RF**	1.022	1.063	1.225	1.254	1.210	0.642	0.625	0.661	0.565	0.600	1.404	1.318	1.783	1.684
**2.5 ng/ml RF**	1.114	1.031	1.083	1.212	1.229	0.581	0.579	0.545	0.540	0.574	1.175	1.234	1.192	1.274
**5 ng/ml RF**	1.033	1.080	1.082	1.210	1.220	0.586	0.580	0.558	0.785	0.801	1.188	1.121	1.089	1.239
**10 ng/ml RF**	1.042	1.020	1.018	1.123	1.176	0.628	0.595	0.600	0.587	0.579	1.186	1.475	1.464	1.338
**20 ng/ml RF**	1.013	1.071	1.026	1.026	1.128	0.574	0.546	0.569	0.581	0.591	0.998	1.023	1.351	1.388
**TII**	0.989	1.034	1.005	0.996	1.078	0.592	0.663	0.582	0.561	0.569	1.634	1.563	1.696	1.638
**TII + 1.25 ng/ml RF**	1.252	1.287	1.063	0.968	1.010	0.617	0.585	0.609	0.578	0.548	1.446	1.340	1.419	1.376
**TII + 2.5 ng/ml RF**	1.296	1.290	1.283	1.019	1.090	0.604	0.572	0.533	0.504	0.461	1.405	1.141	1.204	1.156
**TII + 5 ng/ml RF**	1.244	1.340	1.298	1.301	1.049	0.807	0.716	0.667	0.618	0.662	1.299	1.290	1.178	1.196
**TII + 10 ng/ml RF**	1.318	1.345	1.261	1.149	1.052	0.610	0.587	0.600	0.608	0.581	1.209	1.367	1.045	0.986
**TII + 20 ng/ml RF**	1.419	1.213	1.232	1.081	1.142	0.580	0.575	0.560	0.529	0.583	1.334	1.354	1.327	1.260

EXCLI Journal 2023;22:295-309 – ISSN 1611-2156
Received: December 06, 2022, accepted: February 20, 2023, published: February 24, 2023

Supplementary Table 8: Raw data concerning Figure 3B (cont.)

Treatments	Experiment n. 1					Experiment n. 2					Experiment n. 3			
	Normalized values					Normalized values					Normalized values			
**Control**	96.29	106.09	103.17	105.05	89.41	118.99	98.75	100.31	88.01	93.93	97.55	90.49	107.19	104.76
**1.25 ng/ml RF**	96.29	100.15	115.41	118.15	114.00	100.00	97.35	102.96	88.01	93.46	110.09	103.35	139.81	132.04
**2.5 ng/ml RF**	104.96	97.14	102.04	114.19	115.79	90.50	90.19	84.80	84.12	89.41	92.14	96.77	93.47	99.90
**5 ng/ml RF**	97.32	101.75	101.94	114.00	114.94	91.28	90.35	86.82	122.26	124.75	93.16	87.91	85.40	97.16
**10 ng/ml RF**	98.17	96.10	95.91	105.80	110.80	97.82	92.68	93.46	91.44	90.19	93.00	115.66	114.80	104.92
**20 ng/ml RF**	95.44	100.90	96.66	96.66	106.27	89.41	85.05	88.64	90.50	92.06	78.26	80.22	105.94	108.84
**TII**	93.18	97.42	94.69	93.84	101.56	92.22	103.27	90.66	87.39	88.64	128.12	122.56	132.98	128.44
**TII + 1.25 ng/ml RF**	117.96	121.25	100.15	91.20	95.16	96.11	91.13	94.86	89.93	85.37	113.39	105.08	111.27	107.90
**TII + 2.5 ng/ml RF**	122.10	121.54	120.88	96.01	102.69	94.08	89.00	82.93	78.41	71.72	110.17	89.48	94.41	90.65
**TII + 5 ng/ml RF**	117.20	126.25	122.29	122.57	98.83	125.69	111.52	103.89	96.26	103.11	101.86	101.16	92.38	93.79
**TII + 10 ng/ml RF**	124.18	126.72	118.81	108.25	99.11	95.02	91.44	93.46	94.60	90.40	94.81	107.19	81.95	77.32
**TII + 20 ng/ml RF**	133.69	114.28	116.07	101.85	107.59	90.35	89.57	87.13	82.30	90.71	104.61	106.17	104.06	98.80

EXCLI Journal 2023;22:295-309 – ISSN 1611-2156
Received: December 06, 2022, accepted: February 20, 2023, published: February 24, 2023

Supplementary Table 9: Raw data concerning Figure 3C. C20 cells were treated with RF alone at different concentration and in combination with TII for 16 h. TII consists in a mixture of proinflammatory cytokines, including TNFα, IL-1β and IFNγ, each used at the same concentration (50 pg/ml). After the incubation period, cytotoxicity was assessed by measuring the activity of lactate dehydrogenase (LDH) released in the incubation media. Raw data were collected as Optical Density (OD) at 490 nm (OD_{490}). In the table the net OD_{490} is reported per each sample. It was calculated by subtracting the average OD_{490} values measured for the blanks, *i.e.* incubation medium not exposed to the C20 cells, to the absorbance values measured for each sample. Average OD_{490} for the blanks was 0.138 (± 0.005 SD, n=3) in experiment n. 1; 0.127 (± 0.002 SD, n=2) in Experiment n. 2; and 0.108 (± 0.003 SD, n=3) in experiment n. 3. Data were then normalized as percentage of controls and to generate Figure 3C. Data in bold were considered outliers and excluded from the analysis. RF=remifentanil; TII= mixture of TNFα, IL-1β and IFNγ; OD= Optical Density.

**Treatments**	**Experiment n. 1**					**Experiment n. 2**					**Experiment n. 3**			
	**net OD_{490}**					**net OD_{490}**					**net OD_{490}**			
**Control**	0.022	0.022	0.017	0.027	0.024	ND	ND	0.004	0.023	0.005	0.033	0.020	0.018	0.032
**1.25 ng/ml RF**	0.018	0.021	0.015	0.020	0.020	0.008	0.010	0.015	0.019	0.015	0.046	0.022	0.026	0.011
**2.5 ng/ml RF**	0.028	0.021	0.022	0.026	0.022	0.011	0.020	0.012	0.011	0.015	0.029	0.025	0.016	0.016
**5 ng/ml RF**	0.027	0.018	0.018	0.017	0.052	0.010	0.007	0.004	0.008	0.006	0.028	0.028	0.039	0.021
**10 ng/ml RF**	0.019	0.021	0.020	0.035	0.044	0.008	0.011	0.007	0.010	0.004	0.046	0.030	0.036	0.048
**20 ng/ml RF**	0.047	0.017	0.024	0.025	0.031	0.012	0.006	0.007	0.006	0.007	0.029	0.026	0.025	0.019
**TII**	0.023	0.020	0.020	0.024	0.019	0.010	0.001	0.001	0.004	ND	0.029	0.030	0.022	0.031
**TII + 1.25 ng/ml RF**	0.019	0.021	0.023	0.019	0.018	0.009	0.003	0.001	0.007	0.041	0.025	0.022	0.026	0.037
**TII + 2.5 ng/ml RF**	0.026	0.023	0.026	0.026	0.023	0.012	0.007	0.003	0.011	0.006	0.021	0.030	0.025	0.028
**TII + 5 ng/ml RF**	0.031	0.024	0.019	0.030	0.024	0.013	0.004	0.010	0.009	0.004	0.034	0.033	0.025	0.042
**TII + 10 ng/ml RF**	0.022	0.021	0.021	0.037	0.029	0.015	0.003	0.007	0.008	0.018	0.048	0.033	0.032	0.026
**TII + 20 ng/ml RF**	0.022	0.020	0.019	0.029	0.046	0.068	0.005	0.006	0.004	0.007	0.029	0.032	0.034	0.036

EXCLI Journal 2023;22:295-309 – ISSN 1611-2156
Received: December 06, 2022, accepted: February 20, 2023, published: February 24, 2023

Supplementary Table 9: Raw data concerning Figure 3C (cont.)

Treatments	Experiment n. 1					Experiment n. 2					Experiment n. 3			
	Normalized values					Normalized values					Normalized values			
**Control**	98.21	98.21	75.89	120.54	107.14	ND	ND	37.50	215.63	46.88	128.48	76.54	70.74	124.23
**1.25 ng/ml RF**	80.36	93.75	66.96	89.29	89.29	75.00	93.75	140.63	178.13	140.63	178.60	85.17	102.62	41.65
**2.5 ng/ml RF**	125.00	93.75	98.21	116.07	98.21	103.13	187.50	112.50	103.13	140.63	111.00	96.46	62.92	62.92
**5 ng/ml RF**	120.54	80.36	80.36	75.89	232.14	93.75	65.63	37.50	75.00	56.25	109.77	107.82	149.86	80.02
**10 ng/ml RF**	84.82	93.75	89.29	156.25	196.43	75.00	103.13	65.63	93.75	37.50	177.66	117.32	141.23	186.60
**20 ng/ml RF**	209.82	75.89	107.14	111.61	138.39	112.50	56.25	65.63	56.25	65.63	111.38	101.59	98.90	74.94
**TII**	102.68	89.29	89.29	107.14	84.82	93.75	9.38	9.38	37.50	ND	114.54	115.24	84.91	119.58
**TII + 1.25 ng/ml RF**	84.82	93.75	102.68	84.82	80.36	84.38	28.13	9.38	65.63	**384.38**	96.87	86.12	100.77	143.04
**TII + 2.5 ng/ml RF**	116.07	102.68	116.07	116.07	102.68	112.50	65.63	28.13	103.13	56.25	80.80	114.90	96.11	108.94
**TII + 5 ng/ml RF**	138.39	107.14	84.82	133.93	107.14	121.88	37.50	93.75	84.38	37.50	132.10	126.48	97.10	162.88
**TII + 10 ng/ml RF**	98.21	93.75	93.75	165.18	129.46	140.63	28.13	65.63	75.00	168.75	184.99	126.93	122.43	101.41
**TII + 20 ng/ml RF**	98.21	89.29	84.82	129.46	205.36	**637.50**	46.88	56.25	37.50	65.63	112.70	124.34	133.55	140.62

EXCLI Journal 2023;22:295-309 – ISSN 1611-2156
Received: December 06, 2022, accepted: February 20, 2023, published: February 24, 2023

Supplementary Table 10: Raw data concerning Figure 4A. C20 cells were treated with remifentanil (RF) at different concentrations alone or in combination with 50 pg/ml TII for 6 h. TII consists in a mixture of proinflammatory cytokines, including TNFα, IL-1β and IFNγ, each used at the same concentration (50 pg/ml) in the cocktail. After the incubation period, the concentrations of IL-6 (pg/ml) in the incubation media were measured by a specific ELISA assay. The amount of IL-6 was measured in the incubation media after 6 h. Data are shown as pg/ml and derive from 3 independent experiments, each including 4 biological replicates per treatment.

Treatments	Experiment n. 1				Experiment n. 2				Experiment n. 3			
	IL-6 Concentration (pg/ml)				IL-6 Concentration (pg/ml)				IL-6 Concentration (pg/ml)			
**Control**	9.43	9.61	10.63	8.94	8.25	4.67	3.65	4.17	4.67	3.65	ND	ND
**1.25 ng/ml RF**	4.92	3.82	6.30	5.09	3.91	ND	ND	ND	1.51	ND	ND	1.51
**2.5 ng/ml RF**	4.34	3.91	4.92	5.66	5.98	ND	3.27	ND	ND	ND	ND	3.30
**5 ng/ml RF**	6.14	5.42	4.42	5.01	5.98	ND	ND	ND	ND	ND	5.56	ND
**10 ng/ml RF**	6.14	6.22	5.25	4.76	4.25	2.06	3.81	1.87	1.42	4.03	5.44	ND
**20 ng/ml RF**	5.50	5.90	5.82	5.58	6.62	1.51	ND	ND	1.31	ND	ND	22.64
**TII**	453.73	426.72	427.28	378.09	379.70	236.70	244.88	ND	219	243.25	233.96	ND
**TII + 1.25 ng/ml RF**	391.03	365.79	352.53	388.87	514.39	234.25	238.34	ND	226.79	220.68	244.88	ND
**TII + 2.5 ng/ml RF**	394.83	363.66	368.99	349.37	409.03	ND	217.33	206.04	227.90	240.52	213.40	ND
**TII + 5 ng/ml RF**	473.22	404.10	378.62	379.70	356.23	249.22	ND	222.91	226.79	260.53	255.15	ND
**TII + 10 ng/ml RF**	362.60	363.13	346.74	365.79	398.10	250.84	ND	ND	250.84	174.55	386.82	235.60
**TII + 20 ng/ml RF**	366.85	451.46	365.26	338.87	421.72	245.42	210.58	ND	233.41	239.98	236.15	ND

Received: December 06, 2022, accepted: February 20, 2023, published: February 24, 2023

Supplementary Table 11: Raw data concerning Figure 4B. C20 cells were treated with remifentanil (RF) at different concentrations alone or in combination with 50 pg/ml TII for 4 h. TII consists in a mixture of proinflammatory cytokines, including TNFα, IL-1β and IFNγ, each used at the same concentration (50 pg/ml). After the incubation period, the concentration of MCP-1 (pg/ml) in the incubation media were measured by a specific ELISA assay. The amounts of MCP-1 measured in the incubation media after 6 h are reported. Data are shown as pg/ml and derive from 3 independent experiments, each including 4 biological replicates per treatment.

Treatments	Experiment n. 1				Experiment n. 2				Experiment n. 3			
	MCP-1 concentration (pg/ml)				MCP-1 Concentration (pg/ml)				MCP-1 Concentration (pg/ml)			
**Control**	18.50	32.49	32.69	22.77	57.93	58.84	68.39	63.07	44.57	66.63	33.26	31.57
**1.25 ng/ml RF**	21.01	24.92	39.11	43.85	20.33	44.23	50.93	36.61	25.06	19.63	13.88	11.86
**2.5 ng/ml RF**	10.12	18.04	26.60	21.89	42.15	35.83	45.35	32.29	36.10	36.56	62.46	51.03
**5 ng/ml RF**	12.59	29.48	35.64	37.96	40.07	46.10	41.02	29.89	51.69	35.16	49.48	65.97
**10 ng/ml RF**	ND	21.01	29.48	18.50	35.05	39.49	49.82	34.07	17.31	25.32	22.67	38.19
**20 ng/ml RF**	18.74	37.77	74.49	50.74	42.15	39.49	42.15	33.29	28.37	33.02	48.15	23.48
**TII**	667.51	955.17	908.21	959.42	563.36	603.65	540.41	558.54	470.24	735.65	636.05	768.60
**TII + 1.25 ng/ml RF**	1158.86	909.56	936.95	1205.18	480.30	523.48	449.91	571.11	669.33	614.12	740.07	705.37
**TII + 2.5 ng/ml RF**	791.38	542.31	760.71	815.63	565.29	671.76	687.86	635.11	531.33	722.54	550.16	756.47
**TII + 5 ng/ml RF**	949.53	733.20	867.28	975.18	578.92	483.02	477.59	502.17	645.24	672.05	746.00	647.88
**TII + 10 ng/ml RF**	571.11	1384.26	1096.47	997.08	256.54	465.02	429.69	465.02	483.51	615.39	524.36	546.60
**TII + 20 ng/ml RF**	1080.26	827.98	844.27	934.18	454.34	420.98	405.42	406.28	411.28	609.03	657.20	530.16

EXCLI Journal 2023;22:295-309 – ISSN 1611-2156
Received: December 06, 2022, accepted: February 20, 2023, published: February 24, 2023

Supplementary Table 12: Raw data concerning Figure 4C. C20 cells were treated with remifentanil (RF) at different concentrations alone or in combination with 50 pg/ml TII for 6 h. TII consists in a mixture of proinflammatory cytokines, including TNFα, IL-1β and IFNγ, each used at the same concentration (50 pg/ml). After the incubation period, the concentration of IL-8 (pg/ml) in the incubation media were measured by a specific ELISA assay. The released amount of Il-8 measured in the incubation media after 6 h are reported in the table. Data are shown as pg/ml and derive from 4 independent experiments, each including 4 biological replicates per treatment.

Treatments	Experiment n. 1				Experiment n. 2				Experiment n. 3				Experiment n. 4			
	IL-8 Concentration (pg/ml)				IL-8 Concentration (pg/ml)				IL-8 Concentration (pg/ml)				IL-8 Concentration (pg/ml)			
**Control**	ND	1.50	2.76	ND	4.56	8.88	7.21	4.72	ND	ND	ND	ND	ND	1.62	5.85	ND
**1.25 ng/ml RF**	2.41	4.55	9.35	7.67	2.59	3.00	3.83	3.09	ND	ND	ND	ND	ND	ND	9.11	2.77
**2.5 ng/ml RF**	52.21	7.67	8.37	6.66	5.29	4.40	10.07	2.42	ND	ND	ND	ND	ND	ND	1.36	2.56
**5 ng/ml RF**	10.85	4.23	7.24	7.38	4.32	6.01	4.80	6.01	ND	ND	ND	ND	ND	7.26	10.51	13.53
**10 ng/ml RF**	11.12	8.79	6.51	2.93	6.49	4.64	4.80	88.24	ND	ND	ND	ND	1.40	36.86	3.48	46.19
**20 ng/ml RF**	7.81	6.07	6.51	9.35	75.53	31.03	10.94	82.92	25.99	ND	ND	33.41	60.45	63.57	9.51	183.44
**TII**	1432.55	753.48	769.82	953.64	983.51	1263.95	1161.64	1359.97	1795.62	2479.78	1782.83	4244.294	6428.01	4125.50	6958.49	7113.28
**TII + 1.25 ng/ml RF**	2773.53	829.88	835.06	865.11	1067.60	1136.05	1118.25	1250.97	1823.91	2252.78	2114.88	1981.55	10506.28	4246.80	5035.19	7197.23
**TII + 2.5 ng/ml RF**	2647.09	816.97	865.11	900.90	1241.30	1215.83	1420.32	1327.27	1607.42	1772.62	2084.56	2221.40	3982.67	3139.11	3350.56	3946.11
**TII + 5 ng/ml RF**	2920.45	795.19	1124.55	763.52	3383.39	919.51	972.85	1204.83	2092.81	1997.64	1336.93	2109.35	4195.35	3355.86	5156.83	6948.29
**TII + 10 ng/ml RF**	3080.37	907.59	981.19	945.44	4021.37	1124.16	1045.04	1285.31	2112.112	1749.73	1737.07	1847.18	6428.01	3994.92	8812.63	7186.68
**TII + 20 ng/ml RF**	2717.19	833.77	978.42	933.21	3053.43	1436.71	1334.09	1300.28	4437.917	1989.59	1519.69	2212.89	5989.41	3556.78	6523.19	6024.84

EXCLI Journal 2023;22:295-309 – ISSN 1611-2156
Received: December 06, 2022, accepted: February 20, 2023, published: February 24, 2023

Supplementary Table 13: Raw data concerning Figure 5A. C20 cells were treated with 5 ng/ml remifentanil (RF) alone or in combination with 50 pg/ml TII for 16 h. TII consists in a mixture of proinflammatory cytokines, including TNFα, IL-1β and IFNγ, each used at the same concentration of 50 pg/ml in the cocktail. After the incubation period, the mRNA expression levels of the monocyte chemoattractant protein-1 (MCP-1) were quantified through real-time qPCR using the comparative quantitation method based upon the −ΔΔCt method. Relative mRNA concentrations were calculated from the take-off point of reactions (threshold cycle, Ct) taking the expression level of the Control as Calibrator (Cal.) and the expression level of β-actin as normalizing gene. Ct values were obtained at fix threshold of 0.15, in order to combine data from different experiments. Each sample was assessed in triplicate. Raw data presented in the table include Ct values, Ct average and Ct standard deviation (SD), relative expression to calibrator (Rel. Quant. to Cal.) and melting temperature (Tm) of the amplified product, for both MCP-1 and β-actin mRNA. The last column includes the normalized expression values (Normalized Rel. Quant. to Cal.) for the MCP-1 mRNA used to generate Figure 5A and reported in the graph as fold variation versus Control.

Experiment (number/biological replicate)	Treatment	Technical Replicates	mRNA MCP1					mRNA β-actin (Normalizing gene)					-ΔΔCt
			Ct	Ct Average	Ct SD	Rel. Quant. to Cal.	Tm	Ct	Ct Average	Ct SD	Rel. Quant. to Cal.	Tm	Normalized Rel. Quant. to Cal.
1	Control (16 h)	1	25.19	25.23	0.08	1.03	83.25	16.44	16.80	0.38	1.28	86.85	1.00
		1	25.18	25.23	0.08	1.04	83.25	16.75	16.80	0.38	1.03	86.80	1.01
		1	25.32	25.23	0.08	0.94	83.25	17.20	16.80	0.38	0.76	86.80	0.92
	5 ng/ml RF (16 h)	2	25.24	25.14	0.16	0.99	83.25	16.39	16.38	0.03	1.33	86.85	0.74
		2	25.22	25.14	0.16	1.01	83.60	16.35	16.38	0.03	1.36	86.85	0.75
		2	24.95	25.14	0.16	1.21	83.60	16.40	16.38	0.03	1.32	86.85	0.91
	TII (16 h)	3	18.10	18.14	0.22	140.07	83.85	16.76	16.70	0.05	1.03	86.83	130.94
		3	17.94	18.14	0.22	156.50	83.85	16.67	16.70	0.05	1.09	86.80	146.29
		3	18.37	18.14	0.22	116.16	83.25	16.67	16.70	0.05	1.09	86.80	108.59
	TII + 5 ng/ml RF (16 h)	4	18.58	18.63	0.07	100.43	83.25	17.20	17.24	0.04	0.76	86.85	136.84
		4	18.71	18.63	0.07	91.77	83.25	17.26	17.24	0.04	0.73	86.85	125.05
		4	18.60	18.63	0.07	99.04	83.80	17.27	17.24	0.04	0.72	86.80	134.95

EXCLI Journal 2023;22:295-309 – ISSN 1611-2156
Received: December 06, 2022, accepted: February 20, 2023, published: February 24, 2023

Supplementary Table 13: Raw data concerning Figure 5A (cont.)

Experiment (number/biological replicate)	Treatment	Technical Replicates	mRNA MCP1					mRNA β-actin (Normalizing gene)					-ΔΔCt
			Ct	Ct Average	Ct SD	Rel. Quant. to Cal.	Tm	Ct	Ct Average	Ct SD	Rel. Quant. to Cal.	Tm	Normalized Rel. Quant. to Cal.
2	Control (16 h)	5	**27.19**					**16.65**				86.8	
		5	24.73	24.70	0.05	0.98	83.25	14.71	15.04	0.45	1.25	86.85	0.96
		5	24.67	24.70	0.05	1.02	83.25	15.36	15.04	0.45	0.80	87.3	1.00
	5 ng/ml RF (16 h)	6	24.59	24.90	0.30	1.08	83.2	17.79	17.59	0.28	0.15	87.2	6.28
		6	25.18	24.90	0.30	0.72	83.65	17.39	17.59	0.28	0.20	87.15	4.17
		6	24.93	24.90	0.30	0.85	83.7	**14.51**				87.33	4.96
	TII (16 h)	7	16.89	16.92	0.05	224.41	83.85	15.13	15.25	0.16	0.94	86.85	258.75
		7	16.9	16.92	0.05	222.86	83.83	15.36	15.25	0.16	0.80	86.8	256.96
		7	16.98	16.92	0.05	210.84	83.8	**14.11**				86.8	243.10
	TII + 5 ng/ml RF (16 h)	8	16.78	16.79	0.11	242.19	83.25	15.06	14.61	0.40	0.98	87.2	175.95
		8	16.68	16.79	0.11	259.57	83.7	14.28	14.61	0.40	1.69	86.8	188.58
		8	16.9	16.79	0.11	222.86	83.65	14.49	14.61	0.40	1.46	86.85	161.91
3	Control (16 h)	9	**27.48**					15.89	16.00	0.11	1.08	86.85	
		9	25.99	25.82	0.24	0.89	83.60	16.12	16.00	0.11	0.92	86.85	0.88
		9	25.64	25.82	0.24	1.13	83.60	15.99	16.00	0.11	1.01	86.83	1.13
	5 ng/ml RF (16 h)	10	26.62	26.25	0.32	0.57	83.60	15.68	15.90	0.46	1.25	86.80	0.52
		10	26.04	26.25	0.32	0.86	83.60	15.59	15.90	0.46	1.33	87.28	0.77
		10	26.08	26.25	0.32	0.83	83.60	16.43	15.90	0.46	0.74	87.25	0.75
	TII (16 h)	11	20.73	20.66	0.13	33.94	83.65	16.04	15.97	0.08	0.97	86.85	33.13
		11	20.51	20.66	0.13	39.53	83.60	15.87	15.97	0.08	1.09	86.85	38.58
		11	20.75	20.66	0.13	33.47	83.60	15.99	15.97	0.08	1.01	86.85	32.67
	TII + 5 ng/ml RF (16 h)	12	21.05	20.98	0.23	27.19	83.60	16.86	16.63	0.20	0.55	86.85	41.92
		12	20.73	20.98	0.23	33.94	83.60	16.49	16.63	0.20	0.71	86.83	52.33
		12	21.17	20.98	0.23	25.02	83.60	16.55	16.63	0.20	0.68	86.80	38.57

EXCLI Journal 2023;22:295-309 – ISSN 1611-2156
Received: December 06, 2022, accepted: February 20, 2023, published: February 24, 2023

Supplementary Table 14: Raw data concerning Figure 5B. C20 cells were treated with 5 ng/ml remifentanil (RF) alone or in combination with 50 pg/ml TII for 16 h. TII consists in a mixture of proinflammatory cytokines, including TNFα, IL-1β and IFNγ, each used at the same concentration of 50 pg/ml. After the incubation period, interleukin 8 (IL-8) gene expression was quantified through real-time qPCR using the comparative quantitation method based upon the −ΔΔCt method. Relative mRNA concentrations were calculated from the take-off point of reactions (threshold cycle, Ct) taking the expression level of the Control as Calibrator (Cal.) and the expression level of β-actin as normalizing gene. Ct values were obtained at fix threshold of 0.15, in order to combine data from different experiments. Each sample was assessed in triplicate. Raw data presented in the table include Ct values, Ct average and Ct standard deviation (SD), relative expression to calibrator (Rel. Quant. to Cal.) and melting temperature (Tm) of the amplified product, for both IL-8 and β-actin mRNA. The last column includes the normalized expression values (Normalized Rel. Quant. to Cal.) for the IL-8 mRNA used to generate Figure 5B and reported in the graph as fold variation versus Control.

**Experiment (number/biological replicate)**	**Treatment**	**Technical Replicates**	**mRNA IL-8**					**mRNA β-actin (Normalizing gene)**					**-ΔΔCt**
			**Ct**	**Ct Average**	**Ct SD**	**Rel. Quant. to Cal.**	**Tm**	**Ct**	**Ct Average**	**Ct SD**	**Rel. Quant. to Cal.**	**Tm**	**Normalized Rel. Quant. to Cal.**
**1**	**Control (16 h)**	1	**32.66**				81.25	16.44	16.80	0.38	1.28	86.85	
		1	31.39	31.29	0.15	0.93	81.25	16.75	16.80	0.38	1.03	86.80	0.91
		1	31.18	31.29	0.15	1.08	81.25	17.20	16.80	0.38	0.76	86.80	1.05
	**5 ng/ml RF (16 h)**	2	30.71	30.43	0.24	1.49	81.70	16.39	16.38	0.03	1.33	86.85	1.12
		2	30.26	30.43	0.24	2.03	81.65	16.35	16.38	0.03	1.36	86.85	1.52
		2	30.32	30.43	0.24	1.95	81.65	16.40	16.38	0.03	1.32	86.85	1.46
	**TII (16 h)**	3	19.46	19.28	0.28	3628.10	81.83	16.76	16.70	0.05	1.03	86.83	3391.51
		3	18.96	19.28	0.28	5130.91	81.80	16.67	16.70	0.05	1.09	86.80	4796.33
		3	19.43	19.28	0.28	3704.34	81.80	16.67	16.70	0.05	1.09	86.80	3462.78
	**TII + 5 ng/ml RF (16 h)**	4	19.90	19.90	0.02	2674.40	81.75	17.20	17.24	0.04	0.76	86.85	3644.07
		4	19.88	19.90	0.02	2711.73	81.70	17.26	17.24	0.04	0.73	86.85	3694.94
		4	19.93	19.90	0.02	2619.36	81.70	17.27	17.24	0.04	0.72	86.80	3569.07

EXCLI Journal 2023;22:295-309 – ISSN 1611-2156
Received: December 06, 2022, accepted: February 20, 2023, published: February 24, 2023

Supplementary Table 14: Raw data concerning Figure 5B (cont.)

Experiment (number/biological replicate)	Treatment	Technical Replicates	mRNA IL-8					mRNA β-actin (Normalizing gene)					-ΔΔCt
			Ct	Ct Average	Ct SD	Rel. Quant. to Cal.	Tm	Ct	Ct Average	Ct SD	Rel. Quant. to Cal.	Tm	Normalized Rel. Quant. to Cal.
2	**Control (16 h)**	5	28.12	28.66	0.47	1.45	81.8	**16.65**				86.8	1.42
		5	28.91	28.66	0.47	0.84	81.75	14.71	15.04	0.45	1.25	86.85	0.82
		5	28.95	28.66	0.47	0.82	81.75	15.36	15.04	0.45	0.80	87.3	0.80
	**5 ng/ml RF (16 h)**	6	28.65	28.77	0.12	1.01	81.65	17.79	17.59	0.28	0.15	87.2	5.86
		6	28.77	28.77	0.12	0.93	81.65	17.39	17.59	0.28	0.20	87.15	5.39
		6	28.89	28.77	0.12	0.85	81.6	**14.51**				87.33	4.96
	**TII (16 h)**	7	17.54	17.36	0.24	2225.63	81.83	15.13	15.25	0.16	0.94	86.85	2566.21
		7	17.45	17.36	0.24	2368.90	81.83	15.36	15.25	0.16	0.80	86.8	2731.39
		7	17.09	17.36	0.24	3040.30	81.8	**14.11**				86.8	3505.54
	**TII + 5 ng/ml RF (16 h)**	8	16.62	16.77	0.13	4211.15	81.75	15.06	14.61	0.4	0.98	87.2	3059.34
		8	16.84	16.77	0.13	3615.55	81.75	14.28	14.61	0.4	1.69	86.8	2626.64
		8	16.85	16.77	0.13	3590.58	81.65	14.49	14.61	0.4	1.46	86.85	2608.50
3	**Control (16 h)**	9	**30.99**					15.89	16.00	0.11	1.08	86.85	
		9	29.40	29.26	0.21	0.90	81.30	16.12	16.00	0.11	0.92	86.85	0.90
		9	29.11	29.26	0.21	1.11	81.33	15.99	16.00	0.11	1.01	86.83	1.10
	**5 ng/ml RF (16 h)**	10	30.04	29.68	0.35	0.58	81.30	15.68	15.90	0.46	1.25	86.80	0.52
		10	29.35	29.68	0.35	0.94	81.80	15.59	15.90	0.46	1.33	87.28	0.85
		10	29.65	29.68	0.35	0.76	81.80	16.43	15.90	0.46	0.74	87.25	0.69
	**TII (16 h)**	11	19.78	19.52	0.31	711.64	81.85	16.04	15.97	0.08	0.97	86.85	694.53
		11	19.59	19.52	0.31	811.81	81.83	15.87	15.97	0.08	1.09	86.85	792.29
		11	19.18	19.52	0.31	1078.64	81.83	15.99	15.97	0.08	1.01	86.85	1052.71
	**TII + 5 ng/ml RF (16 h)**	12	19.86	19.78	0.09	673.25	81.85	16.86	16.63	0.20	0.55	86.85	1037.90
		12	19.80	19.78	0.09	701.84	81.85	16.49	16.63	0.20	0.71	86.83	1081.98
		12	19.67	19.78	0.09	768.02	81.85	16.55	16.63	0.20	0.68	86.80	1184.00
